# Proteolytic bacteria expansion during colitis amplifies inflammation through cleavage of the external domain of PAR2

**DOI:** 10.1080/19490976.2024.2387857

**Published:** 2024-08-22

**Authors:** Liam Emile Rondeau, Bruna Barbosa Da Luz, Alba Santiago, Miriam Bermudez-Brito, Amber Hann, Giada De Palma, Jennifer Jury, Xuanyu Wang, Elena Francisca Verdu, Heather Jean Galipeau, Corinne Rolland, Celine Deraison, Wolfram Ruf, Premysl Bercik, Nathalie Vergnolle, Alberto Caminero

**Affiliations:** a Department of Medicine, Farncombe Family Digestive Health Research Institute, McMaster University, Hamilton, ON, Canada; b IRSD, Université de Toulouse, INSERM, INRA, ENVT, UPS, Toulouse, France; c Center for Thrombosis and Hemostasis, Johannes Gutenberg University Medical Center, Mainz, Germany; d Department of Immunology and Microbiology, The Scripps Research Institute, La Jolla, CA, USA

**Keywords:** Inflammatory bowel disease, proteolytic activity, inflammation, colitis, DSS-induced colitis, protease-activated receptor 2 (PAR2), gnotobiotic mice, proteases, microbiota

## Abstract

Imbalances in proteolytic activity have been linked to the development of inflammatory bowel diseases (IBD) and experimental colitis. Proteases in the intestine play important roles in maintaining homeostasis, but exposure of mucosal tissues to excess proteolytic activity can promote pathology through protease-activated receptors (PARs). Previous research implicates microbial proteases in IBD, but the underlying pathways and specific interactions between microbes and PARs remain unclear. In this study, we investigated the role of microbial proteolytic activation of the external domain of PAR2 in intestinal injury using mice expressing PAR2 with a mutated N-terminal external domain that is resistant to canonical activation by proteolytic cleavage. Our findings demonstrate the key role of proteolytic cleavage of the PAR2 external domain in promoting intestinal permeability and inflammation during colitis. In wild-type mice expressing protease-sensitive PAR2, excessive inflammation leads to the expansion of bacterial taxa that cleave the external domain of PAR2, exacerbating colitis severity. In contrast, mice expressing mutated protease-resistant PAR2 exhibit attenuated colitis severity and do not experience the same proteolytic bacterial expansion. Colonization of wild-type mice with proteolytic PAR2-activating *Enterococcus* and *Staphylococcus* worsens colitis severity. Our study identifies a previously unknown interaction between proteolytic bacterial communities, which are shaped by inflammation, and the external domain of PAR2 in colitis. The findings should encourage new therapeutic developments for IBD by targeting excessive PAR2 cleavage by bacterial proteases.

## Introduction

The human gastrointestinal tract is exposed to a variety of proteases that are involved in metabolism, cell signaling, and host defense. While the roles of intestinal host proteases in nutrient digestion have been extensively studied, the functions of other extracellular proteases are being increasingly understood. These proteases, produced by epithelial and immune cells, influence inflammation, wound repair, and infection clearance.^1,2^ However, proteases must be tightly regulated to prevent excessive degradation of host proteins or inappropriate mucosal immune activation. Imbalances in proteolytic activity have been observed in animal models of colitis and in patients with inflammatory bowel disease (IBD).^3,4^ In fact, elevated fecal proteolytic activity often precedes the clinical diagnosis of ulcerative colitis and is associated with the presence of proteolytic bacteria.^5^ A recent study also demonstrated that proteolytic bacteria and their proteases correlate positively with colitis severity.^6^ The intestinal microbiota is indeed an abundant reservoir of proteases, as microbes produce a diversity of proteases for metabolism, defense, and invasion of host tissues.^7^ While proteases that are increased in chronic diseases are mainly of host origin,^8,9^ the role of bacterial proteases in promoting inflammation is emerging.^10,11^

PARs are a family of G-protein-coupled receptors that are widely expressed in the gastrointestinal tract and mediate the proinflammatory and pronociceptive actions of proteases through multiple signaling cascades.^12^ These transmembrane receptors feature an N-terminal extracellular domain and a C-terminal intracellular extremity. The canonical activation of PARs is mediated by proteolytic cleavage of the N-terminal sequence that releases a new motif of amino acids that binds to the transmembrane domain like an agonist.^13^ The N-terminal external domain of PARs can be cleaved by proteases sourced from the circulation (coagulation factors), inflammatory cells (mast cells, neutrophils, and macrophages), epithelial cells, and intestinal microorganisms.^12,14,15^ Notably, PAR2 activation induces a strong inflammatory response,^16^ and has implications in the pathogenesis of several inflammatory and autoimmune disorders.^17^ Its expression spans a variety of human tissues,^18^ including colon, where higher levels are observed in patients with ulcerative colitis.^19^ Studies have shown that PAR2 deficiency protects against inflammation, while its activation influences multiple aspects of the tissue response to injury.^13,20,21^ Importantly, PAR2 interacts with other G-protein coupled receptors, such as other PARs,^22^ and toll-like receptors that are crucial in microbial and immune responses relevant to IBD.^7,23^ Therefore, studies using mice that are entirely PAR2-deficient should be interpreted with caution, and further investigation into the canonical activation of PAR2 in inflammation is required. PAR2 activation is directly achieved by cleavage of its N-terminal external domain by proteases including trypsin, tryptase, and elastase.^24,25^ Early research also indicates microbial proteases that have similar function to the aforementioned enzymes, such as *Porphyromonas gingivalis* proteases or *Pseudomonas aeruginosa* elastase, can activate PAR2 canonically.^26,27^ However, the specific role of the activation of the PAR2 N-terminal external domain by microbial and host proteases in the context of colitis remains incompletely understood.

We investigated the role of cleavage of the external domain of PAR2 and bacterial proteases in colitis using mice harboring intact PAR2 that exhibits a mutated (R38E) N-terminal activation site that is resistant to proteolytic cleavage and subsequent activation by microbial and host enzymes. This provides a valuable approach to study the direct role of proteases on PAR2’s canonical activation while maintaining relevant homeostatic pathways.^22,23,28^ We observed that mice with protease-resistant PAR2 developed attenuated inflammation using two models of colitis, one chemically- and the other hapten-induced. In contrast, wild-type mice presenting protease-sensitive PAR2 developed severe colitis marked by elevated intestinal permeability, pro-inflammatory gene expression, and mucosal damage. Severe inflammation in these mice promoted the expansion of bacteria producing highly active proteases that further cleave the external domain of PAR2. When identical microbial proteolytic stimulus was introduced through germ-free colonization in both mouse strains, the protease-resistant PAR2 mice developed attenuated colitis. Furthermore, proteolytic imbalance induced by gnotobiotic colonization with *Enterococcus* and *Staphylococcus* that express PAR2-cleaving enzymes exacerbated colitis in wild-type mice. Our study identifies a novel colitogenic mechanism by which intestinal injury promotes microbial proteolytic imbalance that exacerbates inflammation via PAR2.

## Materials and methods

### Mice

Specific-pathogen free (SPF) C57BL/6N mice of both sexes were originally purchased from Taconic and subsequently bred and housed at McMaster University’s Central Animal Facility (CAF) under SPF conditions. Protease-resistant PAR2 mutant breeding pairs (R38E-PAR2), on a C57BL/6N background, were originally provided by Dr Wolfram Ruf from Johannes Gutenberg University (Mainz, Germany) and were also bred and housed at McMaster’s CAF under SPF conditions.^28^ R38E-PAR2 mice have an intact PAR2 signaling pathway, but a point mutation, replacing arginine (R) at position 38 with glutamic acid (E) in the N-terminal external domain, prevents cleavage and canonical activation by proteases. Canonical activation of PAR2 requires proteolytic cleavage of the peptide bond between R38 and S39 in the N-terminal external domain, which is modified in this mouse strain. For colonization and gnotobiotic experiments, germ-free C57BL/6N and R38E-PAR2 mice were generated through two-stage embryo transfer and bred under germ-free conditions in the Axenic Gnotobiotic Unit at McMaster University.^10^ All mice had unlimited access to food and water and were kept on a 12-h light/12-h dark cycle. All experiments were conducted with approval from the McMaster University Animal Care Committee and McMaster Animal Research Ethics Board (AREB) in accordance with the Animal Utilization Protocols (AUP#170836 & AUP#190618). Ethical regulations for animal testing and research were strictly followed in the study. Mice were matched by sex and age.

### Overall experimental design

Six-to-eight-week-old wild-type SPF C57BL/6N (wild-type) and SPF R38E-PAR2 mice were used. Intestinal injury and colitis were induced by administering 3.5% dextran sulfate sodium (DSS) or 2.5% trinitrobenzene sulfonic acid (TNBS).

In subsequent experiments, six-to-eight-week-old germ-free wild-type and R38E-PAR2 mice were colonized with microbial communities (ex-germ-free mice) to study the impact of controlled microbiota on proteolytic function and colitis severity. For SPF colonization experiments, germ-free wild-type and R38E-PAR2 mice were colonized by oral gavage with fresh cecal content of an SPF wild-type mouse, using previously described protocols.^5,29^ We also used mice colonized with altered Schaedler flora (ASF),^30^ a well-defined and naïve microbiota with limited proteolytic bacteria, to test the effect of bacterial proteases during intestinal inflammation. To generate ASF mice co-colonized with or without proteolytic bacteria (*Enterococcus faecalis and Staphylococcus epidermidis*), germ-free wild-type mice were colonized with fresh cecal content of an ASF mouse and received an oral gavage with the proteolytic bacteria (10^10^ colony forming units per mouse) or PBS as a control. Colitis was induced with 2.5% DSS as described above.

For assessment of inflammation of germ-free wild-type compared to germ-free R38E-PAR2 mice, DSS (2%) was provided to eight-week-old mice.

The concentration of DSS provided to germ-free and ex-germ-free colonized mice was reduced due to their increased susceptibility to colitis as previously reported.^31^

At sacrifice, colitis severity, intestinal permeability, fecal proteolytic activity, bacterial translocation, intestinal microbiota, and cleavage of the PAR2 N-terminus were assessed.

### Induction and assessment of DSS colitis

Intestinal injury was induced in C57BL/6N and R38E-PAR2 mice with different microbiota backgrounds by administering DSS (36,000–50,000 MW; MP Biomedicals LLC) in drinking water for 5 days, followed by 2 days on normal drinking water before sacrifice. Mice that did not receive DSS were given water. DSS intake was monitored daily throughout all experiments.

Colitis severity was evaluated by measuring colon length, body-weight loss, spleen mass, stool consistency, and stool blood. Stool consistency and blood were each scored on a scale of 0–3, as previously described,^32^ then summed for a stool index.

### Induction and assessment of TNBS colitis

Experimental colitis was induced using TNBS in SPF wild-type and R38E-PAR2 mice, as previously described.^33^ Briefly, the mice were anesthetized with isoflurane (4%). TNBS (2.5% dissolved in 50% ethanol) was administered by rectal insertion of a polyethylene catheter into the colon. Mice were monitored for body-weight loss and overall mortality for 72 h. At the endpoint, the mice were euthanized, and their colons were excised, rinsed with saline, and scored for macroscopic damage, according to the Wallace Scoring system as previously described.^33^ Score: 0 - No damage. 1 - Hyperemia without ulcers. 2 - Hyperemia and thickening of bowel wall without ulcers. 3 - One site of ulceration without bowel wall thickening. 4 - Two or more sites of ulceration or inflammation. 5 - 0.5 cm of inflammation and major damage. 6 to 10 - 1 cm of major damage. The score is increased by one for every 0.5 cm of damage observed, up to a maximum of 10. Colon length and bacterial translocation to the spleen were quantified.

### Evaluation of microscopic damage after colitis

For histologic analysis of DSS and TNBS colitis, tissue samples were collected from the distal colon, placed in 10% formalin and embedded in paraffin. All tissues were then stained with filtered hematoxylin (Sigma‐Aldrich) and 1% eosin Y solution (Sigma‐Aldrich) and visualized for histological evaluation of morphology under light microscopy (Olympus, ON, Canada). Two sections of the distal colon were evaluated for evidence of inflammation by two blinded observers. Microscopic damage on a scale of 0–4 was determined, as previously described.^32,34^

### Intestinal permeability

Colonic paracellular permeability (^51^Cr-EDTA mucosal-to-serosal flux), tissue conductance, and ion secretion were evaluated *ex vivo* using proximal colon sections and the Ussing chamber technique, as previously described.^35^ Proximal colon sections of 1.5 cm were collected, opened along the mesenteric border, and mounted into an Ussing chamber. The chambers exposed 0.25 cm^2^ of tissue surface area to 4 ml of circulating oxygenated Krebs buffer containing 10 mM glucose (serosal side) and 10 mM mannitol (mucosal side) maintained at 37°C aerated with 95% O_2_ and 5% CO_2_. The flux rate of ^51^Cr-EDTA from mucosa to serosa was quantified in samples using a liquid scintillation counter and expressed as % hot sample/h/cm^2^. Ion secretion (expressed as μA/cm^2^) across the epithelium was measured via a short circuit response (Isc, μA) injected through the tissue under voltage-clamp conditions. Tissue conductance (passive permeability to ions, expressed as ms/cm^2^) was calculated using Ohm’s law. Baseline ion secretion and tissue conductance were recorded at equilibrium 20 min after mounting sections. The Ussing chamber system was from Physiologic Instruments (Reno, NV, USA).

### Bacterial translocation

Translocation of live bacteria was evaluated by homogenizing spleen tissue (1:10 w/v) in brain heart infusion (BHI) broth and plating serial dilutions on BHI-agar and MacConkey-agar media under aerobic and anaerobic conditions for 48 h at 37°C. The number of colony forming units (CFU) were counted.

### Pro-inflammatory and barrier gene expression analysis

RNA was extracted using RNA Later (Invitrogen) stabilized colon tissue and the RNeasy Mini Kit (Qiagen). NanoString nCounter gene expression was run according to the manufacturer’s instructions (NanoString Technologies, Seattle, WA) using two panels (Mouse Inflammation Panel, 254 genes; Custom gene panel, 144 genes, Table S1). The results obtained were analyzed with nSolver 2.5 (NanoString Technologies). The raw mRNA counts obtained from the NanoString platform were normalized using background correction, positive control normalization, and housekeeping gene normalization to account for technical variations. The resulting normalized mRNA counts represent the relative expression levels of genes, providing a basis for subsequent statistical analysis. Differentially expressed genes are visualized in heat maps and graphs. For network map visualization, ratios of gene expression (by NanoString nSolver) for wild-type and R38E-PAR2 mice were uploaded into IPA software (Qiagen) for further analysis. The network map is generated using a proprietary algorithm, and the network score is based on the hypergeometric distribution of genes and is calculated with a right-tailed Fisher’s exact test.

### Microbiota analysis

Fecal pellets were collected and flash-frozen on dry ice. DNA was extracted from the samples and the hypervariable V3-V4 regions of the 16S rRNA gene were amplified with polymerase chain reaction (PCR) using Taq polymerase (Life Technologies, Carlsbad, CA), as previously described.^36^ Forward barcoded primers targeting the V3 region (v3f_341f-CCTACGGGNGGCWGCAG) and reverse primers targeting the V4 region (v4r_806r-GGACTACNVGGGTWTCTAAT) were used. Forward primers included six-base pair barcodes to allow multiplexing samples. Purified PCR products were sequenced using the Illumina MiSeq platform by the McMaster Genomics facility. Primers were trimmed from the obtained sequences with Cutadapt software,^37^ and processed with Divisive Amplicon Denoising Algorithm 2 (DADA2; version 1.14.0) using the trained SILVA reference database (version 138.1).^38^ A phylogenetic tree of the sequences was calculated using FastTree 2,^39^ and the data was explored using the phyloseq package (version 1.30.0) in R (version 3.6.2).^40^ After data cleanup, a total of 1,625,464 reads were obtained with a minimum of 17,764 and a maximum of 103,644 with an average of 41,002 reads per sample. Alpha-diversity was measured using observed species. Beta-diversity was calculated on normalized data using the Bray-Curtis dissimilarity method. The originated matrix was ordinated using non-metric multidimensional scaling (NMDS). Differences between bacterial communities were tested by permutational multivariate analysis of variance (PERMANOVA) using the vegan package (version 2.5.6) for R.

### Proteolytic activities

Non-specific proteolytic activities (total and overall), elastase-like, and trypsin-like proteolytic activities were measured in fecal samples and in bacterial culture supernatants. Non-specific proteolytic activities were measured using substrates azocasein (total) and gluten (overall) that can be cleaved by various proteases and therefore assess broad-spectrum protease activity. These assays enable detection of both bacterial and host protease activities that have similar function.

Elastase-like activity was analyzed using Suc-Ala3-pNA (Sigma-Aldrich), a substrate commonly used to measure elastase activity, including that of neutrophil, pancreatic, and bacterial enzymes. This substrate is highly specific due to its sequence of alanine residues, which these enzymes cleave. The cleavage of Suc-Ala3-pNA by elastase-like enzymes releases the chromogenic p-nitroanilide, which is measured spectrophotometrically. Total proteolytic activity was analyzed using azocasein (Sigma-Aldrich), a nonspecific substrate that releases an azo dye upon cleavage, also measured spectrophotometrically. Briefly, fecal and bacterial supernatants were incubated with each substrate in 50 mM Tris-HCl buffer (pH 8.2) supplemented with 1 mM CaCl2, 50 mM NaCl, and 0.25% Triton at 37°C. Absorbance was measured at various time points. Enzyme units were determined using standard curves of elastase from porcine pancreas (Sigma-Aldrich) for elastase-like activity and trypsin (Sigma-Aldrich) for total proteolytic activity.

Trypsin-like activity of fecal and bacterial supernatants was determined using the Trypsin Activity Assay Kit (Abcam, Cambridge, UK) following the manufacturer’s recommendations.

Overall proteolytic activity of fecal sample supernatants was determined with a bioassay using agar plates enriched with gluten (1%; Sigma-Aldrich), in which samples were incubated for 24 h. Overall proteolytic activity was quantified by measuring the diameter of the halo surrounding the inoculation site on the gluten plate.^10^

### Isolation and identification of proteolytic bacteria

Mouse fecal samples were plated on selective agar media plates designed to isolate proteolytic bacteria. The following culture media containing different protein sources were used: (1) Gluten agar (1%) and BHI + gluten (1%) agar; (2) Elastin agar (0.5%; elastin from bovine neck ligament; Sigma-Aldrich) and BHI + elastin (0.5%) agar and; (3) Casein agar (1%; Sigma-Aldrich) and BHI + casein (1%) agar. Plates were incubated with fecal samples for 48 h in anaerobic conditions (Bactron IV anaerobic chamber) and a total of 95–100 bacterial isolates per sample were randomly selected after incubation. All bacterial isolates were streaked on the substrate-containing agar plates and grown in liquid OptiMEM reduced serum medium (Gibco) at 37°C for 48 h. Positive proteolytic activity was determined by the presence of a halo surrounding the inoculation site on substrate-containing media. Elastase activity was analyzed by incubating bacterial supernatants grown in OptiMEM with FITC-elastin (AnaSpec) kinetically for 1 h. To confirm the presence of proteolytic bacteria in feces of mice, fecal homogenates were plated on gluten agar (1%) and the presence of zones of gluten clearance was observed. Bacteria with proteolytic activity were identified using Sanger technology. Briefly, DNA from isolates was extracted by picking single bacterial colonies into water, boiling, and centrifuging at 10,000 RPM for 2 min. The 8F-926 R region of the 16S rRNA gene was amplified by PCR using the extracted DNA, and sequences were determined using Sanger sequencing (FW primer: 5’- AGAGTTTGATCCTGGCTCAG-3’, RV primer: 5’-CCGTCAATTCCT-TTRAGTTT-3’). The resulting sequences for the isolates were taxonomically assigned using the NCBI nucleotide collection database.^41^

### Cleavage of the PAR2 external domain by bacteria

Chinese hamster ovary (CHO) cells, in which NanoLuc luciferase (Nluc) is inserted at the PAR2 amino terminus, were used to measure PAR2 external domain cleavage. Briefly, the Nluc reporter tag was cloned in frame with the human form of PAR2 complementary DNA and its stop codon was mutated to insert an eYFP tag (Nluc-hPAR2-eYFP). A stable CHO cell line was developed using positive selection with geneticin antibiotic (G418). The cell line was routinely grown in Ham’s F-12 medium supplemented with 10% fetal bovine serum, and 0.8 mg/ml G418 plasmocin (Invitrogen/ThermoFisher, Carlsbad, CA) on Nunclon Delta Surface Cell Culture Flasks (Sigma-Aldrich, St. Louis, MO) at 5% CO_2_ in a 37°C humidified incubator. Then, 1.5 × 10^4^ cells were plated into each well of a 96-well black plate for 24 h. The cell monolayers were then washed three times with Hank’s balanced salt solution (pH 7.4; Thermofisher), and 100 ul of OptiMEM was added to each well and cultured for approximately 16 h. Bacteria were incubated for 16 h in OptiMEM at 37°C, after which culture medium (20 μl) was added to the Nluc-expressing cell monolayers (final volume 120 μl) and incubated for 15 min at 37°C. Supernatant was collected and centrifuged for 5 min at 12,000 rpm. Then, 80 μl of supernatant was transferred to a 96-multiwell white plate followed by the addition of 80 μl of 50-fold diluted luciferase assay substrate solution (Promega Corporation, Madison, WI). The released luciferase activity was measured using a Varioskan plate reader (ThermoFisher) in duplicate. Trypsin was used as a positive control.

### Goblet cell determination

Goblet cells were visualized using Alcian Blue.^42^ Briefly, for acid mucin staining, sections were deparaffinized, hydrated, and stained with Alcian Blue (Sigma-Aldrich) in 3% acetic acid (pH 2.5) for 30 min, then washed in running water for 5 min. The slides were counterstained with hematoxylin (Sigma-Aldrich) for 20 s and dehydrated, cleared, and mounted with Permount mounting media. The mucin-like glycoproteins stained with Alcian Blue were quantified by counting the number of goblet cells in 10 randomly chosen crypts in the distal colon.

### Statistical analysis

All variables were analyzed with SPSS version 18.0 (SPSS Inc., USA) and GraphPad Prism 9 (GraphPad Software, USA). Categorical variables are expressed as numbers and percentages, and quantitative variables as means ± standard error of the mean (s.e.m.) or medians with interquartile range for parametric or nonparametric data, respectively. Normal distribution was determined by D’Agostino – Pearson omnibus normality test, Shapiro – Wilk test, and Kolmogorov – Smirnov test with Dallal-Wilkinson-Lillie correction. Data are depicted as dot plots, with each dot representing an individual mouse, or biological replicate. The one-way analysis of variance (ANOVA) test was used to evaluate differences between more than two groups with a parametric distribution and Tukey’s post-hoc correction was applied. Student’s *t*-test (two-tailed) was performed to evaluate the differences between two independent groups or paired samples as appropriate. Data with non-normal distribution were evaluated with Kruskal – Wallis test for more than two groups, and Mann – Whitney test for two independent groups. Wald test was used for statistical analysis of gene expression by Nanostring nSolver 2.5. To compare paired samples where the data is not normally distributed, Wilcoxon signed-rank test was used. A *p* value < 0.05 was selected to reject the null hypothesis by two-tailed tests. Information regarding specific *p* values, value of *n*, and how data are presented can be found in figure legends.

## Results

### Functional external domain of PAR2 regulates barrier function during colitis

**Figure 1.**
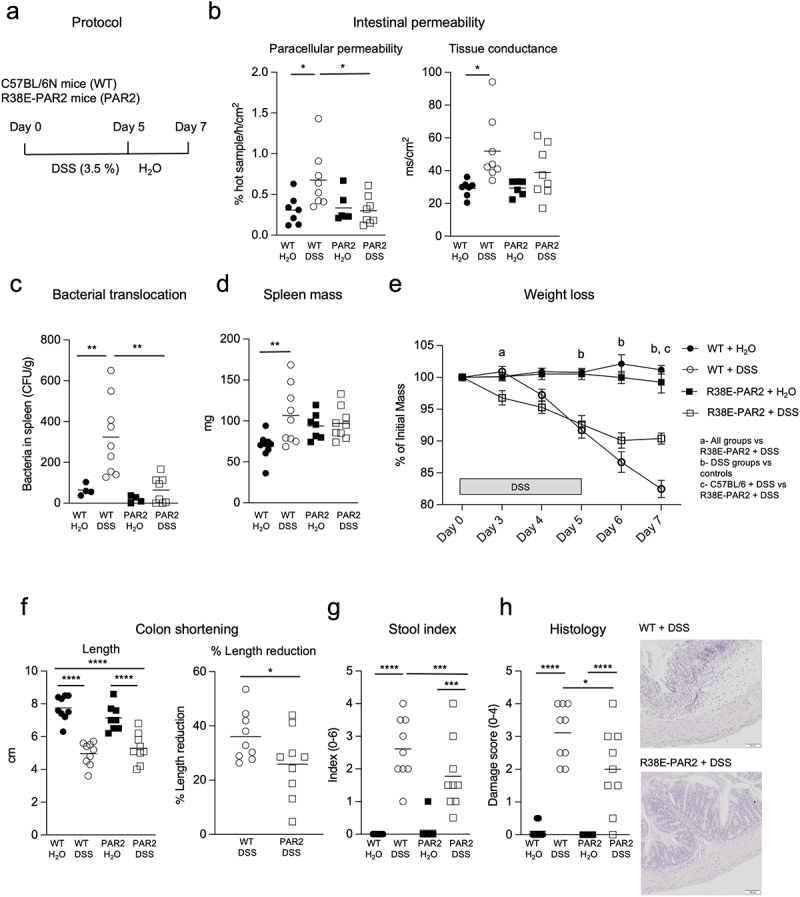
PAR2 external domain activation increases intestinal permeability and determines colitis severity. (a)- experimental design. SPF wild-type and protease-resistant PAR2 mutant (R38E-PAR2) mice were given 3.5% DSS in drinking water for 5 days followed by water for the next 2 days. Control mice received only water during the entire procedure. (b)- colonic intestinal barrier function represented as paracellular permeability (% hot sample/h/cm^2^)and tissue conductance (ms/cm^2^) of mice after DSS or water control (n = 6-8). (c)- bacterial translocation into the spleen (CFU/g) of mice after DSS colitis or water control (n = 4-9). (d)- spleen mass (mg) of mice after DSS colitis or water control (n = 7-9). (e)- weight loss during DSS colitis or water control (n = 8-9). (f)- colon length of mice after DSS colitis or water control (n = 8-9). Percent colon length reduction relative to water treated controls is shown for each mouse strain. (n = 8-9). (g)- stool index scored on a scale of 0-6 of mice after DSS colitis or water control (n = 8-9). (h)- microscopic injury scoring of the colonic mucosa of mice after DSS colitis or water control (n = 8-9). One representative image of the colon is shown for wild-type and R38E-PAR2 mice after DSS. Data are presented as mean where each dot represents an individual mouse (a-d, f-h) or where each dot represent the mean ± s.e.m. of the group (e). Displayed *p* values were calculated by a one-way analysis of variance (ANOVA) with Tukey’s post-hoc test. *p < 0.05, **p < 0.01, ***p < 0.001, ****p < 0.001.

Intestinal inflammation induced by DSS resulted in increased colonic paracellular permeability and tissue conductance in wild-type mice compared to water-treated controls that did not receive DSS. This increase was not observed in R38E-PAR2 mice, which exhibited significantly reduced paracellular permeability compared to wild-type mice. These findings suggest that a functional PAR2 external domain is essential for regulating intestinal permeability during colitis (Figures 1(a,b)). Additionally, there were no differences in ion secretion observed before or after colitis in either mouse strain (Supplementary Figure S1). We next investigated systemic bacterial escape by measuring the translocation of live bacteria to the spleen. While total bacterial counts in the spleen of wild-type mice with colitis were higher than water-treated controls, R38E-PAR2 mice with colitis had no significant bacterial translocation (Figure 1(c)). An increase in spleen mass, indicative of a systemic inflammatory response, was observed in wild-type but not R38E-PAR2 mice after colitis (Figure 1(d)). These results suggest that activation of the PAR2 pathway is involved in the regulation of barrier function during colitis.

### PAR2 external domain determines colitis severity

The key role of PAR2 activation in the progression of inflammation during colitis is further highlighted by the protection afforded to protease-resistant R38E-PAR2 mice. Colitis development reduced the body mass of both wild-type and R38E-PAR2 mice, however R38E-PAR2 mice were protected from the severe weight loss experienced by wild-type mice (Figure 1(e)). Relative to water treated controls, R38E-PAR2 mice experienced significantly less reduction in colon length compared to wild-type mice after colitis, suggesting attenuated tissue damage (Figure 1(f)). Colon length reduction of wild-type and R38E-PAR2 mice was calculated because we have previously observed shorter colons in SPF R38E-PAR2 mice without colitis. R38E-PAR2 mice were also protected from the development of severe diarrhea and stool blood exhibited in wild-type mice (Figure 1(g)). Histologic evaluation of colitis severity revealed that R38E-PAR2 mice had significantly less colonic inflammatory cell infiltration and architectural damage than wild-type mice after colitis (Figure 1(h)). We confirmed our findings that R38E-PAR2 mice develop attenuated inflammation and bacterial translocation compared to wild-type mice using the TNBS hapten-induced colitis model (Supplementary Figure S2). These results indicate that the canonical activation site of PAR2 is an important signaling mechanism in the development of colitis.

### PAR2 external domain mediates pro-inflammatory gene expression during colitis

**Figure 2.**
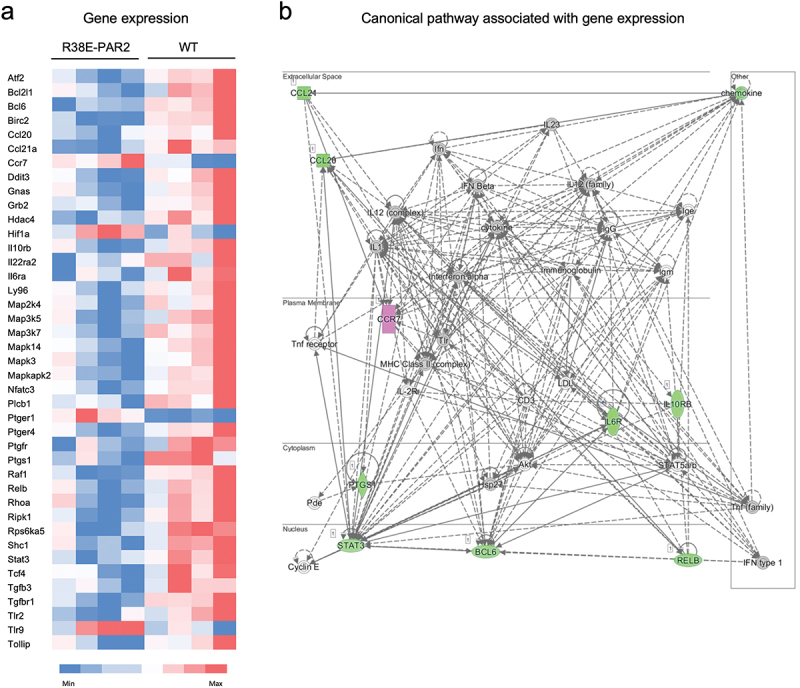
Pro-inflammatory genes are upregulated in wild-type, but not R38-PAR2 mice, after colitis. (a)- heat map of gene expression in the colon of wild-type and R38E-PAR2 mice after DSS colitis. Only genes differentially expressed between groups are shown (*N* = 4). P-values were calculated by nSolver 2.5 using the Wald test. (b)- canonical pathways dysregulated in wild-type mice compared to R38E-PAR2 mice after dss-induced colitis associated with the “digestive system development and function” network, generated by ingenuity pathway analysis software. The colors in the heat map (a) are based on up-regulated (red) and down-regulated (blue) genes in the colon (n = 4), while the colors in the network (b) represent up-regulated (pink) and down-regulated (green) genes in the colon of R38E-PAR2 samples (n = 4).

To investigate the underlying pathways, we measured transcript levels of 254 genes in the colon of wild-type and R38E-PAR2 mice after colitis. We observed that 37 proinflammatory genes were down-regulated and 4 were up-regulated in R38E-PAR2 mice compared with wild-type mice after colitis (Figure 2(a)). These include genes downstream of PAR2 activation, such as those mediating mitogen-activated protein kinases (*Map2k4, Map3k5, Map3k7, Mapk14* and *Mapkapk12*), NF-kappa-B (*Relb, Ripk1* and *Hif1a*), prostaglandin E (*Pteger1* and *Pteger4*), and Rho (*Rhoa*) signaling. Additionally, some genes were associated with other G-protein coupled receptors (*Ccr7* and *Gnas*), and pathways that communicate with PAR2, such as toll-like receptor signaling (*Tlr2, Tlr9, Ly96*, and *Rps6ka5*). Wild-type mice also had higher expression of genes associated with inflammatory cytokines (*Il6* and *Il22*) and transcription factors downstream of both toll-like receptor and PAR2 signaling (*Stat3* and *Bcl6*), compared to R38E-PAR2 mice after colitis.

The transcripts differentially expressed after colitis in wild-type mice were associated with functional networks including “Digestive System Development and Function” (Figure 2(b)) and “Organismal Injury and Cell Death” (Supplementary Figure S3). The latter functional network involves the G beta-gamma complex, found downstream in the G protein-coupled receptor signaling pathway. These results suggest that colitis leads to the expression of pro-inflammatory genes through PAR2 external domain cleavage, which participates in intestinal injury and gut dysfunction development.

### Functional PAR2 dictates microbiome changes after colitis

**Figure 3.**
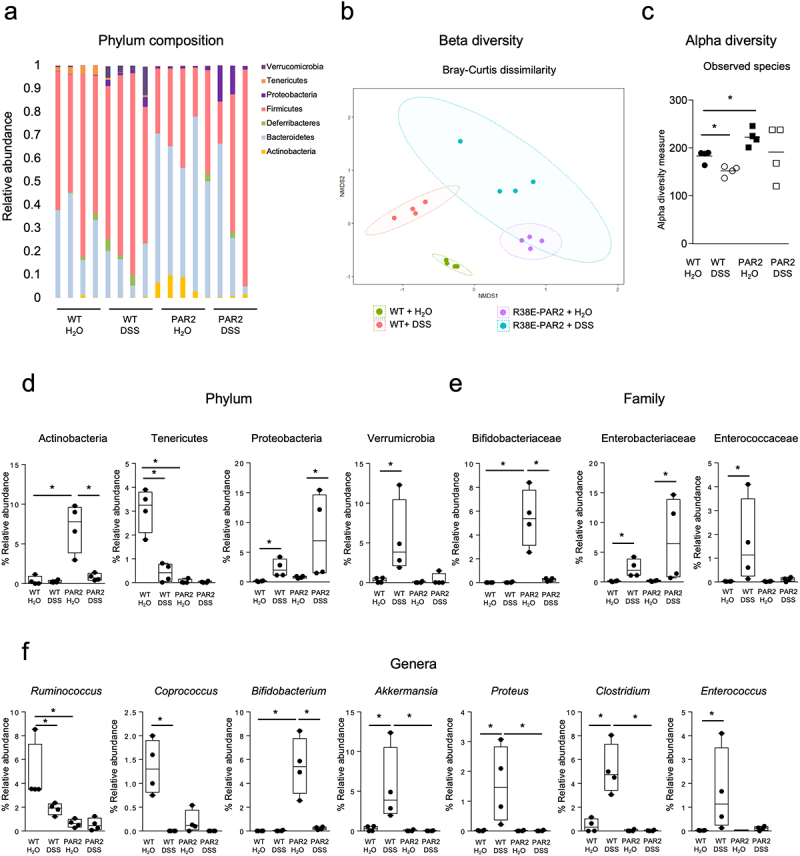
Different fecal microbiota based on colitis and mouse strain. (a)- phylum-level relative abundance of the microbial composition in wild-type and R38E-PAR2 mice after DSS colitis or water control. Each bar represents an individual mouse. (b)- Non-metric multidimensional scaling (NMDS) plot of fecal microbiota of mice after DSS colitis or water control based on Bray–Curtis dissimilarity matrix (beta-diversity). (c)- observed species (alpha-diversity) in fecal microbiota of mice after DSS colitis or water control. (d-f)- significantly altered taxa in fecal microbiota of wild-type and R38E-PAR2 mice after DSS colitis or water control at the phylum (d), family (e) and genus (f) levels. Each dot represents an individual mouse. Data are presented as median (c) or median with interquartile range and whiskers extending from minimum to maximum (d-f). Significance was determined using Mann–Whitney and Kruskal–Wallis test. Displayed *p* values survived 5% (d) and 10% (e, f) false discovery rate (FDR) correction. (n = 4). * p < 0.05, ** p < 0.01, *** p < 0.001, **** p < 0.001.

To investigate the interaction between the external domain of PAR2 and microbes, we examined the fecal microbiota of wild-type and R38E-PAR2 mice with and without colitis. Wild-type and R38E-PAR2 mice differed in microbial profiles, independent of colitis (Figures 3(a,b)). Colitis led to changes in the dissimilarity of bacterial communities (beta-diversity) in both mouse strains. However, further taxonomic separation (Figure 3(b)) and a reduction in the number of observed bacterial species (alpha-diversity; Figure 3(c)) were observed in wild-type mice which experienced more severe colitis compared to R38E-PAR2 mice.

Colitis induced bacterial taxonomic changes in both mouse strains (Figure 3(d)). Phyla such as Actinobacteria and Tenericutes, were lower after colitis in R38E-PAR2 and wild-type mice, respectively. In contrast, phyla associated with inflammatory capacity such as Proteobacteria and Verrucomicrobia were increased after colitis in both mouse strains (Figure 3(d)). At the Family level, R38E-PAR2 mice had a lower abundance of *Bifidobacteriaceae* and a higher abundance of *Enterobacteriaceae* after colitis. This increase of *Enterobacteriaceae* was also observed in wild-type mice, together with the proliferation of *Enterococcaceae* (Figure 3(e)). Colitis was also linked to lower abundance of anaerobic commensal genera of the colon, including *Rumminococcus* and *Coprococcus* in wild-type mice, and *Bifidobacterium* in R38E-PAR2 mice. Interestingly, the proliferation of classic proteolytic bacterial groups including *Enterococcus*, *Clostridium*, and *Proteus* was observed in wild-type, but not R38E-PAR2 mice after colitis (Figure 3(f)). Altogether, these results suggest that colitis induces shifts in the fecal microbiota that are unique to each mouse strain and are determined by the functional activity of PAR2. However, whether this microbial diversification involves a functional proteolytic change that contributed to colitis severity was unclear.

### Colitis increases bacterial proteolytic activity and expands PAR2 cleaving bacteria

**Figure 4.**
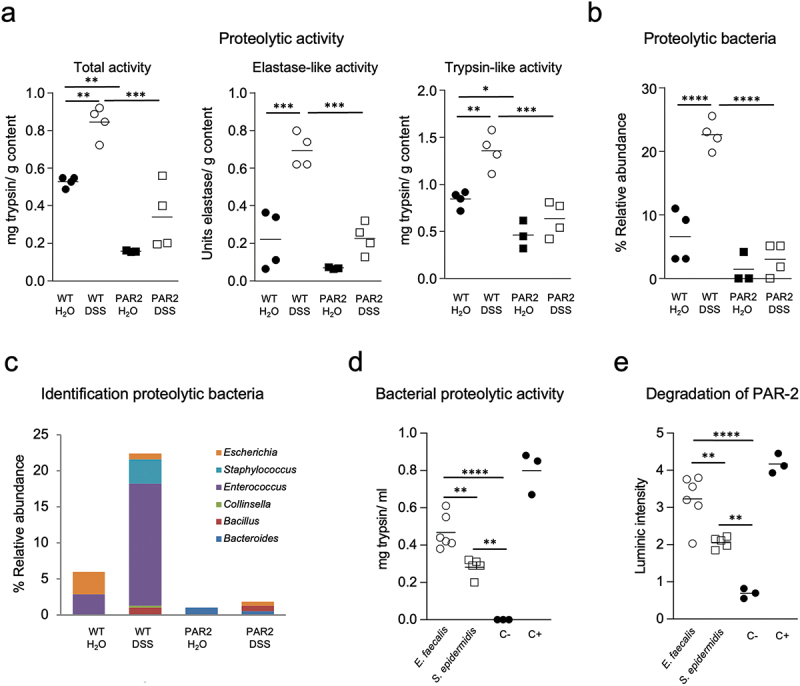
Proteolytic activity and bacteria increase after colitis in wild-type mice. (a)- fecal proteolytic activity of wild-type and R38E-PAR2 mice after DSS colitis or water control. Total proteolytic activity, elastase-like activity, and trypsin-like activity were measured in fecal samples. (b)- relative abundance (%) of proteolytic bacteria isolated from fecal samples of mice after DSS colitis or water control using protein-enriched agar media. (c)- average relative abundance (%) of proteolytic bacteria among the total isolated fecal bacteria per group (n = 3-4). (d)- proteolytic capacity of *Enterococcus faecalis* and *Staphylococcus epidermidis* strains isolated from fecal samples of wild-type mice after DSS. (e)- capacity of *E. faecalis* and *S. epidermidis* strains isolated from fecal samples of wild-type mice after DSS to degrade the external domain of PAR2. Data are presented as mean where each dot represents an individual mouse (a-c) or one biological replicate (d-e). Displayed *p* values were calculated by a one-way analysis of variance (ANOVA) with Tukey’s post-hoc test. * p < 0.05, ** p < 0.01, *** p < 0.001, **** p < 0.0001.

As PAR2 is activated by proteases, we measured total fecal proteolytic activity, elastase-like, and trypsin-like activity in wild-type and R38E-PAR2 mice with or without colitis. Wild-type mice had higher total proteolytic, elastase-like, and trypsin-like activity than R38E-PAR2 mice, regardless of whether they had colitis (Figure 4(a)). Colitis was associated with increased proteolytic activities in wild-type mice, but there was only a mild, non-significant increase in total proteolytic activity and elastase-like activity, but not trypsin-like activity, in R38E-PAR2 mice with colitis. The observed increase in proteolytic activity associated with colitis was less pronounced in R38E-PAR2 mice compared to wild-type mice (Figure 4(a)), which was similarly observed in TNBS colitis (Supplementary Figure S2).

While increased host proteases have been previously reported in animal models of colitis,^43^ the role of bacterial proteases has been underestimated. Based on the observed expansion of classic proteolytic taxa after colitis (Figure 3(f)), we aimed to characterize the main proteolytic bacteria. Fecal bacteria were isolated from wild-type and R38E-PAR2 mice with or without colitis. Culture-based and proteolytic activity assays revealed an expansion of proteolytic taxa in wild-type but not in R38E-PAR2 mice after colitis (Figure 4(b)). The main proteolytic bacterial groups increased in wild-type mice after colitis are *Enterococcus* and *Staphylococcus* (Figure 4(c)). Given that the observed proliferation of *Enterococcus* is consistent with the 16S data by Illumina (Figure 3(f)), we explored this genus further in the context of PAR2. Proteolytic activity determinations confirmed the high proteolytic capacity of *Enterococcus faecalis* and *Staphylococcus epidermidis* strains isolated from wild-type mice after colitis (Figure 4(d)). Both bacterial taxa had the capacity to degrade the external domain of PAR2 *in vitro* (Figure 4(e)). These results suggest that under inflammatory conditions, microbial proteolytic activity with the capacity to activate PAR2 is enhanced.

### Proteolytic microbiota worsens colitis in mice with protease sensitive PAR2

**Figure 5.**
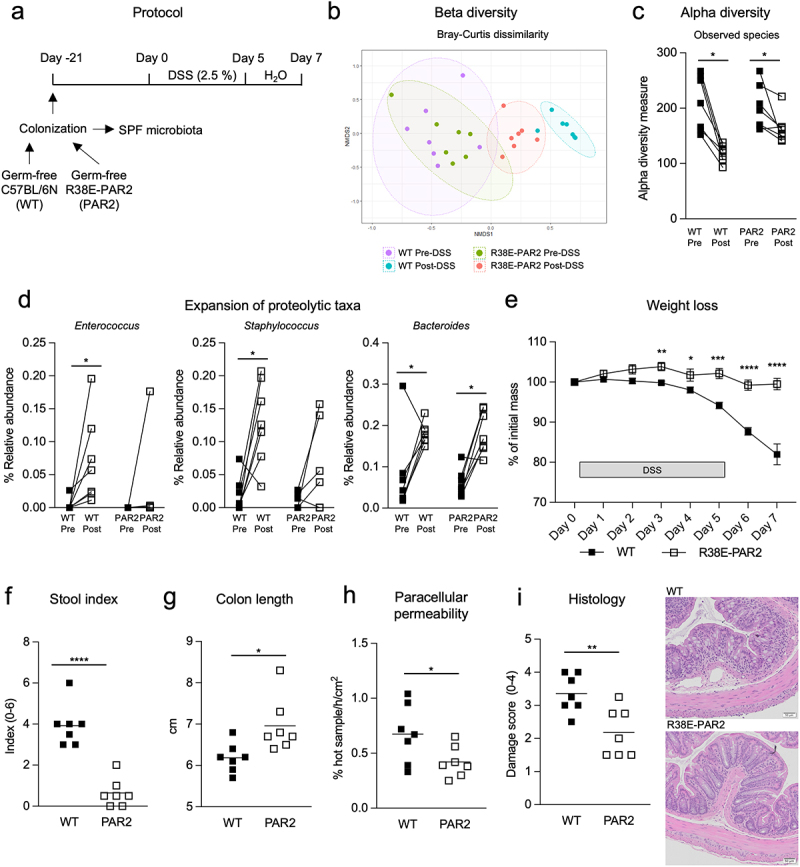
Functional external domain of PAR2 promotes inflammation during colitis when microbial proteolytic stimulus is normalized. (a)- experimental design. Germ-free wild-type and R38E-PAR2 mice were colonized with SPF microbiota from fecal samples of an SPF mouse. After three weeks of microbiota stabilization, mice were given 2.5% DSS in drinking water for 5 days followed by water for the next 2 days (n = 7). (b)- Non-metric multidimensional scaling (NMDS) plot of fecal microbiota of wild-type and R38E-PAR2 pre- and post- DSS colitis based on Bray–Curtis dissimilarity matrix (beta-diversity). (c)- observed species (alpha-diversity) in fecal microbiota of mice pre- and post- DSS colitis. (d)- relative abundance of proteolytic taxa in fecal microbiota of mice pre- and post- DSS colitis. (e)- weight loss during DSS colitis. (f)- stool index scored on a scale of 0-6 after DSS colitis. (g)- colon length of mice after DSS colitis. (h)- colonic intestinal barrier function represented as paracellular permeability (% hot sample/h/cm^2^) of mice after DSS. (i)- microscopic injury scoring of the colonic mucosa of mice after DSS colitis. One representative image of the distal colon is shown for each group. Data are presented as individual dots representing each mouse where the lines indicate paired samples (c-d), mean ± s.e.m. (e), or mean where each dot represents an individual mouse (f-i). Displayed *p* values were calculated by a paired Wilcoxon test (C-D) or Student’s t-test (E-I). * p < 0.05, ** p < 0.01, *** p < 0.001, **** p < 0.001.

To further test the role of proteolytic cleavage of PAR2 in inflammation under similar microbial and proteolytic conditions, we generated germ-free R38E-PAR2 mice. There were no differences in overall intestinal physiology, barrier function, or colonic inflammatory or barrier genes between germ-free wild-type and R38E-PAR2 mice (Supplementary Figure S4 and Supplementary Table S1). Germ-free wild-type and R38E-PAR2 mice also develop similar inflammation during DSS colitis in the absence of microbes (Supplementary Figure S5), underscoring the role of microbes in PAR2-mediated worsening of colitis. Next, we colonized germ-free wild-type and R38E-PAR2 mice with cecal samples from SPF mice harboring microbiota with high proteolytic capacity, then induced colitis with DSS (Figure 5(a)). After colonization, the SPF microbial proteolytic activity phenotype was transferred to both wild-type and R38E-PAR2 mice (Supplementary Figure S6). Beta-diversity revealed that wild-type and R38E-PAR2 mice were colonized similarly (Figure 5(b)). Consistent with naturally colonized mice (Figure 3(b)), a more pronounced microbial shift was observed in wild-type compared to R38E-PAR2 mice after colitis (Figure 5(b)). Both mouse strains had similar alpha-diversity, which reduced after colitis (Figure 5(c)). Colitis expanded taxa known to be proteolytic including *Enterococcus* and *Staphylococcus* in wild-type mice, and to a lesser degree in R38E-PAR2 mice. *Bacteroides*, a genus with known proteolytic capacity,^5^ expanded in both wild-type and R38E-PAR2 mice (Figure 5(d), Supplementary Figure S7). Total proteolytic activity was increased in both mouse strains after inflammation (Supplementary Figure S6). Elastase-like and trypsin-like activity were only significantly increased in wild-type mice (Supplementary Figure S6). R38E-PAR2 mice developed attenuated weight loss, bloody and soft stool, and colon shortening compared to wild-type mice (Figure 5(e–g)). Compared to R38E-PAR2 mice, wild-type mice had higher paracellular permeability and histologic damage after colitis (Figure 5(h–i)). There were no differences in tissue conductance or ion secretion between the mouse strains (Supplementary Figure S8A-B). Despite the increase in paracellular permeability of wild-type mice, we did not observe any differentially expressed genes related to barrier function, including tight junction proteins, cadherins, claudins, occludin, and mucins, which parallel the results in tissue conductance (Supplementary Figure S9). However, we did observe higher expression of key inflammatory cytokines (*Il6, Il1b, Il17b*) and chemokines (*Cxcl1* and *Cxcl2*) involved in colitis in wild-type compared to R38E-PAR2 mice. Wild-type mice also had larger spleens compared to R38E-PAR2 mice, which is indicative of systemic inflammation (Supplementary Figure S8C). Collectively, these findings indicate that cleavage of PAR2’s external domain is a key event in the inflammatory response during colitis, even when controlling for microbial proteolytic stimulus.

### Expanded proteolytic bacteria exacerbate intestinal inflammation

**Figure 6.**
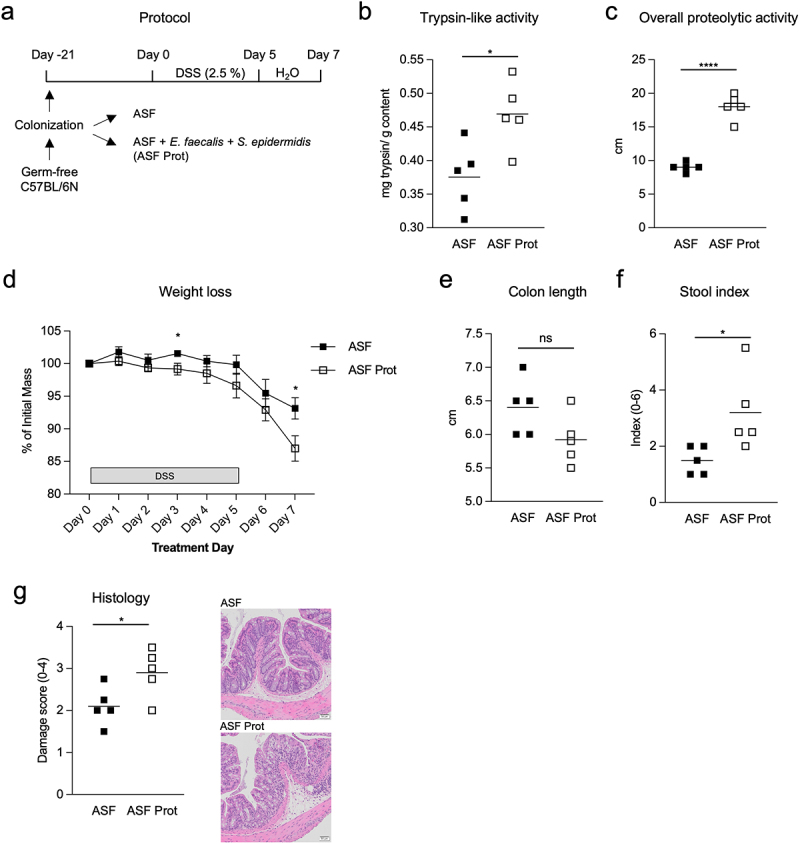
Expanded proteolytic taxa enhance proteolytic activity and promote inflammation in gnotobiotic mice. (a)- experimental design. Germ-free wild-type mice were colonized with ASF or co-colonized with ASF and *E. faecalis* and *S. epidermidis* (ASF prot). After three weeks, colitis was induced with 2.5% DSS in drinking water for 5 days followed by water for the next 2 days (n = 5). (b)- trypsin-like activity of fecal samples. (c)- overall fecal proteolytic activity (diameter of halo surrounding fecal inoculation site in gluten agar). (d)- weight loss during DSS colitis. (e)- colon length after DSS colitis. (f)- stool index scored on a scale of 0-6 after DSS colitis. (g)- microscopic injury scoring of the colonic mucosa of mice after DSS colitis. One representative image of the distal colon is shown for each group. Data are presented as mean where each dot represents an individual mouse (A, B, C, E, F, G) or mean ± s.e.m. (D). Displayed *p* values were calculated by a one-way analysis of variance (ANOVA) with Tukey’s post-hoc test. * p < 0.05, ** p < 0.01, *** p < 0.001, **** p < 0.001.

We next studied the colitogenic effects of the proteolytic bacteria *Enterococcus* and *Staphylococcus* that expanded during colitis in wild-type mice. We colonized germ-free wild-type mice with either clean ASF microbiota alone or in combination with proteolytic *E. faecalis* and *S. epidermidis* isolated from feces of wild-type SPF mice after colitis (Figure 6(a)). Compared with ASF-colonized mice, ASF mice co-colonized with proteolytic bacteria showed higher trypsin-like activity (Figure 6(b)) and overall proteolytic activity (Figure 6(c) and Supplementary Figure S10A). Culture of fecal pellets confirmed the presence of bacteria releasing proteases in mice colonized with the proteolytic bacteria (Supplementary Figure S10B). Mice colonized with the proteolytic strains exhibited more weight loss than ASF controls after colitis (Figure 6(d)). Additionally, colonization with the proteolytic bacteria was associated with a mild, though not statistically significant, reduction in colon length (Figure 6E), and an increased stool index (Figure 6(f)). Histologic analysis of the distal colon revealed greater tissue damage in mice colonized with proteolytic microbiota (Figure 6(g)). These findings support that the presence of proteolytic *E. faecalis* and *S. epidermidis* strains that expand during intestinal inflammation can exacerbate colitis severity in wild-type gnotobiotic mice, potentially through activation of the PAR2 pathway.

## Discussion

There is growing evidence that proteolytic imbalance is associated with functional and inflammatory gastrointestinal diseases.^5,44^ To develop new preventive and therapeutic approaches, the main triggers of inflammation and the signaling events that contribute to disease onset and progression must be understood. Our study provides compelling evidence for the critical role of PAR2 external domain cleavage and further activation by proteolytic microbes in driving the inflammatory process during colitis. Moreover, our findings reveal that the expansion of PAR2-cleaving proteolytic opportunistic pathogens during inflammation may further contribute to the exacerbation of inflammation through PAR2 activation. These results point to a dual implication of PAR2 in both host and microbial signaling in the context of mucosal inflammation.

PARs are activated when their N-terminal extracellular domain is cleaved by proteases, releasing a new motif of amino acids that binds to the receptor like an agonist and triggers intracellular-signaling events.^25^ The activation of the PAR family (PAR1, 2, 3 and 4) is protease-specific, tightly regulated, and influences physiological functions in the gut, such as motility, permeability, and nociception. Proteases such as trypsin, tryptase, elastase, and microbial proteases with similar structures and functions activate PAR2 by proteolytic cleavage. An imbalance in these proteases can lead to dysregulated PAR2 signaling, resulting in a variety of pro-inflammatory, pronociceptive, and proliferative effects. This dysregulation is particularly relevant for PAR2, which has been implicated in the pathogenesis of intestinal inflammation.^19,36,45^ Unfortunately, mechanistic studies using experimental models of colitis have produced conflicting results due to the pro-inflammatory and anti-inflammatory properties of this receptor.^46–48^ Hyun et al. showed that PAR2-deficient mice have reduced inflammation in three different models of colitis, including DSS and TNBS.^13^ However, the use of PAR2-deficient models limits the understanding of the molecular mechanisms related to the activation site and proteolytic-specific activation in the context of colitis. Indeed, PAR2 has many functions that are unrelated to receptor cleavage/activation, such as serving as a co-receptor for other proteolytically cleaved PARs and interacting with receptors involved in microbial signaling such as toll-like receptors, which are also linked to IBD. In the present study, we used R38E-PAR2 mice on a C57BL/6N background presenting an N-terminal external domain lacking the canonical activation site. This mutation prevents activation by proteolytic cleavage and mimics some aspects of PAR2 knock-outs,^49^ but leaves many of the other relevant functions of PAR2 intact.^22,28^ Thus, our findings using this model establish the pivotal molecular mechanism by which proteolytic activation of the PAR2 external domain drives the inflammatory response underlying colitis.

We induced acute intestinal injury in R38E-PAR2 and wild-type mice using one cycle of DSS in drinking water. Wild-type mice experienced progressive and more severe weight loss, losing 20% of initial mass by the end of the experiment. Colitis also increased intestinal permeability, bacterial translocation, and led to spleen enlargement in wild-type mice, but this was not observed in R38E-PAR2 mice. This is consistent with previous studies showing that PAR2 activation increases paracellular permeability in the colon and promotes bacterial translocation into peritoneal organs.^20^ Finally, histologic analysis revealed that R38-PAR2 mice developed attenuated inflammation and colon tissue damage compared to wild-type mice. We observed similar protection from inflammation in R38E-PAR2 mice during TNBS hapten-induced colitis. Together, these results demonstrate the significant influence of PAR2 activation by proteolytic cleavage of its external domain in the inflammatory process in colitis.

To investigate how PAR2 external domain cleavage exacerbates colitis, we quantified colonic transcript levels of pro-inflammatory genes after colitis induction. Many genes were up-regulated in wild-type mice but not in R38E-PAR2 mice, including genes relevant to PAR2 signaling. PAR2 activation induces the assembly of a MAPK signaling module through a mechanism that depends on β-arrestins.^50,51^ Several genes associated with MAPK were down-regulated in R38E-PAR2 mice compared to wild-type mice including *Map2k4, Map3k5, Map3k7, Mapk14* and *Mapkapk12*. PAR2 activation is also suspected to involve the Rho family of proteins which are important in various cellular processes.^52–54^ The gene *Rhoa* of this family was upregulated in wild-type but not R38E-PAR2 mice after colitis. PAR2 has also been shown to mediate the activation of NF-kappa-B and the release of prostaglandin E,^55,56^ which are key mediators in immune responses in preclinical models of colitis and IBD.^57–60^ Genes associated with NF-kappa-B activation, such as *Melb, Ripk1* and *Hif1a*,^61–63^ or with receptors of prostaglandin E (*Pteger1* and *Pteger4*) were altered in wild-type mice compared to R38E-PAR2 mice after colitis. Additionally, genes related to other receptors involved in IBD, such as toll-like receptors (*Tlr2, Tlr9, Ly96* and *Rps6ka5*), were also differentially expressed between the two mouse strains. Toll-like receptor signaling participates in innate host defense in the intestine, mucosal homeostasis, and microbial recognition.^64^ There is evidence of communication between PAR2 and toll-like receptors,^23,28^ as well as other G-protein couple receptors altered in our study (*Ccr7* and *Gnas*).^65^ The overactivation of both toll-like receptors and/or PARs has been linked to increased pro-inflammatory responses involving NF-kappa-B and other pro-inflammatory mediators such as IL-6, IL-22, and STAT-3.^66–72^ These are classic immune mediators involved in IBD and genes directly encoding them were upregulated after colitis in wild-type mice with protease-sensitive PAR2.^73–75^ Overall, these results suggest that inflammation during experimental colitis leads to increased expression of pro-inflammatory genes relevant to intestinal injury and gut dysfunction development through proteolytic PAR2 activation.

Increased proteolytic activity in fecal samples and colonic biopsies of patients with IBD has been previously demonstrated.^3,5,9,76^ Similarly, preclinical models of colitis also exhibit an imbalance in proteolytic activity associated with inflammation.^8^ In this study, we found that colitis significantly increased total proteolytic activity, elastase-like, and trypsin-like activity in wild-type mice. Although R38E-PAR2 mice also had a trend for higher total proteolytic activity and elastase-like activity after colitis, no differences were observed for trypsin-like activity. Previous research has shown that increased proteolytic activity in IBD is often due to host proteases, such as neutrophil elastase, cathepsin G, and thrombin, which are released by infiltrating immune cells or the mucosa.^8,9,77^ Despite this, the role of bacterial proteases in IBD is being established,^7^ and the specific mechanisms underlying their colitogenic effects have received insufficient attention.

Functional and compositional microbial changes have been described in IBD patients and preclinical mouse models. Here, we found that colitis-induced changes in the fecal microbiota of both wild-type and R38E-PAR2 mice. However, these changes differed between mouse strains, in part due to their different original microbiota. Classic anaerobic members of the colonic microbiota were reduced in both wild-type (*Rumminococcus* and *Coprococcus*) and R38E-PAR2 (*Bifidobacterium*) mice after colitis. In addition, classic proteolytic bacterial groups such as *Enterococcus*, *Clostridium*, and *Proteus* proliferated in wild-type mice after colitis. To better understand the functional attributes of the bacterial expansion after colitis, we isolated fecal bacteria and characterized their proteolytic capacities. We found that the abundance of proteolytic bacteria, specifically *Enterococcus* and *Staphylococcus*, was significantly higher in wild-type mice compared to R38E-PAR2 mice after colitis. The isolated strains of both *E. faecalis* and *S. epidermidis* were able to cleave the external domain of PAR2. Our findings suggest that expansion of proteolytic microbes during intestinal inflammation may contribute to proteolytic imbalance, leading to subsequent cleavage of PAR2 that further incites inflammation. Whether this expansion results from the pro-inflammatory cascade initiated by PAR2 canonical activation by proteases, or some other PAR2-mediated mechanism, remains unclear.

Inflammatory outcomes during colitis are dependent on intestinal microbiota,^5,36^ physiology, and immunity. Indeed, we found that R38E-PAR2 mice exhibit similar intestinal physiology and structure, goblet cell levels, barrier function, and intestinal gene expression to wild-type mice in germ-free conditions. To confirm that the different inflammatory responses observed in the two mouse strains during colitis were not due to different intestinal microbiota, we colonized germ-free wild-type and R38E-PAR2 mice with a standardized SPF microbiota that included proteolytic bacteria. We found that when both mouse strains were exposed to the same proteolytic and microbial conditions, R38E-PAR2 mice developed attenuated colitis compared to wild-type mice. Like prior experiments, we observed that expansion of classic proteolytic bacteria including *Enterococcus*, *Staphylococcus*, and *Bacteroides* occurs during inflammation in wild-type, but not R38E-PAR2 mice. While paracellular permeability was higher in wild-type compared to R38E-PAR2 mice after colitis, no differences in the expression of key genes involved in barrier function were observed. In contrast, when colitis was induced in both mouse strains under germ-free conditions, the absence of any proteolytic microbial stimulus rendered equal inflammatory states. Together, these results confirm that proteolytic cleavage of the PAR2 external domain constitutes a key event in intestinal inflammation, and that microbial stimulus is required. Furthermore, the expansion of proteolytic bacteria is associated with inflammation and may further exacerbate colitis by cleavage of the PAR2 external domain and potentially through other mechanisms.^11,78^

Previous work by others demonstrates that *E. faecalis* isolated from IBD patients produces the extracellular protease gelatinase E, which compromises the epithelial barrier and may contribute to the development of intestinal inflammation in mice through PAR2 canonical activation.^78^ Gelatinase E is a trypsin-like protease that also degrades E-cadherin leading to the loss of barrier function, which further facilitates bacterial invasion, enhances intestinal permeability, and worsens colitis severity independently of PAR2.^78^ Furthermore, extracellular gelatinase E produced by *E. faecalis* increases permeability of epithelial cell monolayers and the colonic epithelium of mice through PAR2.^11^ *Staphylococcus* also secretes trypsin-like extracellular proteases with the capacity to activate PAR2.^79–81^ Similarly, elevated bacterial proteolytic activity has been shown in *Bacteroides* and linked to the development of ulcerative colitis.^5^ In agreement, when we colonized ASF mice with proteolytic *E. faecalis* and *S. epidermidis* that activate PAR2 *in vitro*, these strains elevated fecal trypsin-like and overall proteolytic activities, and worsened colitis severity. The results of the gnotobiotic work demonstrate that proteolytic microbes that cleave the PAR2 N-terminus are colitogenic. Furthermore, they are supported by a recent study showing that Crohn’s disease microbiota worsens experimental colitis through PAR2.^36^

Despite the difficulty in distinguishing host proteases from microbial proteases in animal models, our study supports the role of microbes and their proteases in the observed phenotypes. This is evidenced by our colonization of ASF gnotobiotic mice, which lack proteolytic taxa, with *E. faecalis* and *S. epidermidis*, as well as the absence of differences in colitis severity between wild-type and R38E-PAR2 mice under germ-free conditions. Future studies involving proteomic techniques and genetic manipulation of specific microbial strains could provide deeper insights into the role of microbial proteases in this work. Of note, our study was not designed to properly address sex variations associated with PAR2 activation and intestinal inflammation. Additionally, while the findings herein using the R38E-PAR2 mouse model underscore the significance of microbial proteolytic activation of PAR2 in colitis, further investigations using models of spontaneous colitis are warranted to validate the findings in a broader context of intestinal inflammation.

Building on prior research implicating bacterial proteases in IBD,^5–7,36^ our study provides new evidence on the molecular mechanism and pivotal role that proteolytic bacteria play in activating PAR2 through cleavage of its external domain. The expansion of proteolytic microbes may be a consequence of the inflammatory environment, possibly driven by changes in barrier function, inflammation, or microbiota composition alterations. Indeed, proteases produced by opportunistic pathogens are important for intestinal colonization, competition against commensals, and/or escape from host immune responses.^7^ Our findings suggest that the activation of PAR2 by microbial proteases exacerbates colitis through a feedback loop. In this scenario, inflammation expands proteolytic bacteria, which in turn leads to further cleavage and activation of PAR2. This process perpetuates inflammation and contributes to the pro-inflammatory status and intestinal permeability associated with colitis. The identification of this mechanism suggests that research should be conducted to develop treatments for IBD patients that prevent excessive PAR2 cleavage by bacterial proteases.

## Supplementary Material

Supplemental Material

## Data Availability

All sequencing data have been deposited in the Sequence Read Archive. 16s rRNA gene sequencing data used in this study can be accessed under BioProject ID PRJNA989619 at http://www.ncbi.nlm.nih.gov/bioproject/989619.
