# Supplementary material for: Proteolytic bacteria expansion during colitis amplifies inflammation through cleavage of the external domain of PAR2
Source: Gut Microbes. 2024 Aug 22;16(1):2387857. doi: 10.1080/19490976.2024.2387857 (PMC11346554; doi:10.1080/19490976.2024.2387857)
Supplement: Supplemental Material [file KGMI_A_2387857_SM7854.zip › Supplementary materials/Rondeau Table S1.docx]

| **Supplementary Table 1. Colonic gene expression of germ-free wild-type and R38E-PAR2 mice.** | | | | |
| --- | --- | --- | --- | --- |
| **GENE EXPRESSION** | **Colonic tissue** | | |  |
|  | **WT** |  | **PAR** | ***p*-value** |
| *5ht* | 365.22 ± 43.65 |  | 367.46 ± 48.15 | 0.9937528 |
| *Actb* | 151957.32 ± 2335.05 |  | 150070.28 ± 5522.44 | 0.7308978 |
| *Arg1* | 29.09 ± 1.95 |  | 31.44 ± 4.06 | 0.6974054 |
| *Asic3* | 18.96 ± 1.72 |  | 21.45 ± 3.31 | 0.6288451 |
| *Atp5f1* | 15173.74 ± 547.82 |  | 15222.81 ± 523.61 | 0.9458141 |
| *Bdkrb1* | 16.85 ± 1.21 |  | 21.14 ± 2.2 | 0.1531095 |
| *Bdkrb2* | 208.23 ± 67.52 |  | 239.17 ± 79.39 | 0.8581508 |
| *Bdnf* | 10.42 ± 2.32 |  | 12.44 ± 1.45 | 0.3359997 |
| *Bmp2* | 4606.64 ± 635.51 |  | 5121.46 ± 752.18 | 0.6824520 |
| *C3* | 2900.52 ± 644.1 |  | 2587.91 ± 396.24 | 0.7288811 |
| *Cacna1a* | 132.31 ± 14.37 |  | 121.48 ± 11.21 | 0.6572510 |
| *Cacna1b* | 166.67 ± 20.74 |  | 157.73 ± 22.24 | 0.7650998 |
| *Cacna1h* | 155.93 ± 36.27 |  | 145.47 ± 24.1 | 0.8916098 |
| *Calb* | 7.9 ± 0.51 |  | 9.37 ± 2.33 | 0.7199138 |
| *Calret* | 21345.85 ± 1573.91 |  | 20157.25 ± 897.9 | 0.5724788 |
| *Casp8* | 991.61 ± 34.29 |  | 985.19 ± 25.5 | 0.9030444 |
| *Ccl5* | 291.31 ± 38.27 |  | 228.08 ± 38.63 | 0.2665370 |
| *Ccr2* | 109.92 ± 10.92 |  | 99.15 ± 17 | 0.5090827 |
| *Ccr6* | 178.8 ± 42.02 |  | 108.58 ± 23.64 | 0.2325071 |
| *Cd11b* | 75.19 ± 24.92 |  | 62.76 ± 12.01 | 0.8658339 |
| *Cd11c* | 101.83 ± 13.31 |  | 87.46 ± 19.21 | 0.4680960 |
| *Cd3g* | 70.27 ± 7.38 |  | 53.09 ± 9.44 | 0.1823015 |
| *Cd86* | 45.17 ± 8 |  | 40.19 ± 9.33 | 0.6181590 |
| *Cdh1* | 20556.98 ± 1108.87 |  | 21530.81 ± 1234.44 | 0.6009936 |
| *Cgrp* | 8.68 ± 0.89 |  | 8.49 ± 0.6 | 0.9238753 |
| *Chat* | 33.34 ± 3.9 |  | 31.71 ± 3.09 | 0.8151534 |
| *Cldn1* | 43.02 ± 2.63 |  | 42.75 ± 6.76 | 0.7692131 |
| *Cldn2* | 3147.14 ± 451.5 |  | 3258.77 ± 510.4 | 0.9240001 |
| *Cnlp* | 21.75 ± 1.67 |  | 23.22 ± 1.85 | 0.5665464 |
| *Cnr1* | 146.03 ± 12.54 |  | 134.9 ± 22.31 | 0.5580078 |
| *Cxcl1* | 26.47 ± 6.43 |  | 19.45 ± 1.45 | 0.3879683 |
| *Cxcl2* | 17.62 ± 1.73 |  | 16.69 ± 1.62 | 0.7246655 |
| *Cxcr3* | 41.76 ± 1.56 |  | 36.8 ± 4.41 | 0.2524021 |
| *Cxcr4* | 428.6 ± 190.59 |  | 223.63 ± 36.52 | 0.3570357 |
| *Defcr1* | 11.94 ± 2.23 |  | 12.09 ± 1.54 | 0.8547648 |
| *F2rl1* | 3687.92 ± 353.68 |  | 4115.51 ± 226.55 | 0.3055294 |
| *Gabaa* | 9 ± 1.34 |  | 8.87 ± 0.64 | 0.9287769 |
| *Gabab* | 262.41 ± 47.6 |  | 212.55 ± 31.69 | 0.4303340 |
| *Gapdh* | 4055.72 ± 61.19 |  | 4126.69 ± 161.9 | 0.7308866 |
| *Gata-3* | 43.3 ± 9.28 |  | 37.84 ± 5.52 | 0.7362216 |
| *Gdnf* | 42.25 ± 10.02 |  | 47.74 ± 11.63 | 0.7765793 |
| *Gfap* | 40.65 ± 5.62 |  | 46.77 ± 6.5 | 0.5250654 |
| *Gpr44* | 15.5 ± 1.37 ***** |  | 20.03 ± 1.23 | 0.0405096 |
| *Gusb* | 495.72 ± 37.45 |  | 440.97 ± 45.87 | 0.3600816 |
| *H2-Ab1* | 7744.76 ± 790.12 |  | 6179.96 ± 1087.22 | 0.3134144 |
| *Hif1a* | 4485.36 ± 742.76 |  | 4208.6 ± 800.72 | 0.7110260 |
| *Hmgb1* | 1857 ± 105.1 |  | 1750.59 ± 91.71 | 0.4716555 |
| *Hprt* | 3682.19 ± 108.71 |  | 3668.93 ± 161.53 | 0.9145978 |
| *Hrh1* | 15.89 ± 1.3 |  | 16.05 ± 2.21 | 0.9315019 |
| *Hrh2* | 31.42 ± 1.94 |  | 22.13 ± 4.44 | 0.1208584 |
| *Hrh3* | 15.44 ± 1.08 |  | 13.93 ± 1.18 | 0.3664285 |
| *Hrh4* | 16.53 ± 2.07 |  | 13.43 ± 2.34 | 0.2921292 |
| *Htr1b* | 21.17 ± 2.07 |  | 21.44 ± 3.03 | 0.9234399 |
| *Htr1d* | 32.78 ± 2.75 |  | 27.21 ± 2.49 | 0.1734245 |
| *Htr2b* | 93.98 ± 8 |  | 75.46 ± 5.35 | 0.0910830 |
| *Htr3a* | 232.36 ± 19.83 |  | 217.09 ± 28.82 | 0.6042321 |
| *Htr3b* | 36.71 ± 2.31 |  | 36.24 ± 3.05 | 0.8685543 |
| *Htr4* | 807.39 ± 79.24 |  | 844.08 ± 125.37 | 0.9268218 |
| *Htr7* | 55.53 ± 5.22 |  | 64.3 ± 5.96 | 0.3101831 |
| *I-Fabp* | 5920.93 ± 1123.44 |  | 9637.6 ± 5029.89 | 0.7054118 |
| *Il10* | 18.69 ± 1.4 |  | 20.08 ± 3.57 | 0.9829893 |
| *Il17a* | 8.29 ± 0.61 |  | 8.81 ± 1.04 | 0.7899582 |
| *Il17ra* | 723.7 ± 74.86 |  | 649.66 ± 41.94 | 0.4507932 |
| *Il1b* | 72.62 ± 7.39 |  | 74.44 ± 14.67 | 0.8760822 |
| *Il22ra2* | 752.56 ± 96.78 |  | 560.92 ± 107.51 | 0.2448582 |
| *Il23r* | 24.66 ± 5.51 |  | 26.19 ± 3.58 | 0.7080299 |
| *Il6* | 34.74 ± 1.61 |  | 43.97 ± 4.28 | 0.0865423 |
| *Kcna2* | 52.26 ± 12.5 |  | 54.79 ± 12.25 | 0.9021830 |
| *Kcnk2* | 39.7 ± 4.24 |  | 46.18 ± 6.93 | 0.5463998 |
| *Kit* | 1653.21 ± 219.27 |  | 1701.81 ± 215.8 | 0.8705083 |
| *Lpar1* | 1666.45 ± 95.4 |  | 1669.77 ± 162.85 | 0.9257812 |
| *Lpar2* | 30.65 ± 3.03 |  | 31.61 ± 4.15 | 0.9496862 |
| *Lpar3* | 24.64 ± 2.79 |  | 26.03 ± 2.87 | 0.7286130 |
| *Lpar5* | 1047.38 ± 67.32 |  | 989.4 ± 77.09 | 0.5559996 |
| *Lyz1* | 7659.83 ± 619.39 |  | 5639.38 ± 1291.85 | 0.1998456 |
| *Mapk1* | 4118.52 ± 254.33 |  | 3851.3 ± 235.97 | 0.4611491 |
| *Mmp7* | 42.78 ± 2.81 |  | 51.23 ± 9.68 | 0.6590774 |
| *Mmp9* | 54.39 ± 8.56 |  | 53.5 ± 7.74 | 0.9814735 |
| *Mrc1* | 281.4 ± 59.41 |  | 253.38 ± 58.83 | 0.7219442 |
| *Mrgpra1* | 21.68 ± 1.03 |  | 18.96 ± 2.92 | 0.3395996 |
| *Mrgprb2* | 13.33 ± 1.2 |  | 12.89 ± 1.68 | 0.7642504 |
| *Mrgprf* | 120.64 ± 31.65 |  | 118.08 ± 25.06 | 0.9861596 |
| *Muc2* | 39479.82 ± 5436.57 |  | 37303.68 ± 10094.75 | 0.6585093 |
| *Myd88* | 619.2 ± 21.15 |  | 661.5 ± 23.15 | 0.2269699 |
| *Mylk3* | 19.9 ± 1.73 |  | 22.9 ± 1.48 | 0.2241516 |
| *Nfat* | 1624.03 ± 106.9 |  | 1541.91 ± 87.26 | 0.5797594 |
| *Nfkb1* | 1406.44 ± 81.12 |  | 1284.53 ± 69.9 | 0.2827311 |
| *Nod2* | 28.26 ± 1.85 |  | 28.42 ± 4.95 | 0.7597417 |
| *Nos* | 181.42 ± 22.88 |  | 177.93 ± 25.49 | 0.8642152 |
| *Nos2* | 66.52 ± 5.91 |  | 61.95 ± 7.88 | 0.5804620 |
| *Npy* | 13.87 ± 2.28 |  | 13.96 ± 2.26 | 0.9372219 |
| *Nr2b* | 10.98 ± 1.62 |  | 10.58 ± 2 | 0.8239256 |
| *Nr2d* | 71.85 ± 4.51 |  | 59.21 ± 5.88 | 0.1483937 |
| *Ntrk1* | 9.83 ± 0.98 |  | 9.72 ± 1.11 | 0.9130893 |
| *Ntrk2* | 31.75 ± 2.84 |  | 39.15 ± 15.11 | 0.9760609 |
| *Ntrk3* | 19.56 ± 1.82 |  | 17.39 ± 3.98 | 0.4441841 |
| *Ocln* | 5949.82 ± 436.24 |  | 6696.36 ± 489.26 | 0.3269121 |
| *Oprm1* | 28.97 ± 2.46 |  | 32.65 ± 5.41 | 0.6634597 |
| *P2rx3* | 22.06 ± 3.23 |  | 21.45 ± 4.08 | 0.7885373 |
| *P75* | 130.25 ± 13.22 |  | 120.63 ± 14.61 | 0.6325893 |
| *Pgk1* | 4019.41 ± 74.66 |  | 3790.51 ± 251.06 | 0.3951150 |
| *Pgp9.5* | 7.87 ± 0.52 |  | 7.81 ± 0.68 | 0.8881462 |
| *Piezo1* | 240.87 ± 27.45 |  | 206.34 ± 26.3 | 0.4225637 |
| *Piezo2* | 93.03 ± 13.53 |  | 86.44 ± 12.57 | 0.7239038 |
| *Pparg* | 36.55 ± 1.76 |  | 37.04 ± 4.36 | 0.9265304 |
| *Prdm12* | 9.49 ± 0.82 |  | 8.67 ± 1.06 | 0.4997013 |
| *Rae1* | 768.39 ± 49.09 |  | 705.46 ± 57.68 | 0.4124911 |
| *Ramp1* | 2397.75 ± 128.84 |  | 2591.42 ± 127.25 | 0.3210506 |
| *Reg3g* | 129.44 ± 15.37 |  | 105.3 ± 16.72 | 0.2856188 |
| *Rer1* | 5624.67 ± 188.8 |  | 5828.82 ± 142.32 | 0.4067127 |
| *Rp113a* | 2251.63 ± 136.89 |  | 2284.58 ± 106.51 | 0.8265858 |
| *Rpl27* | 25.91 ± 1.81 |  | 26.73 ± 3.2 | 0.9399986 |
| *Rpl6* | 34471.56 ± 1734.83 |  | 33580.35 ± 2130.48 | 0.7296923 |
| *Rps18* | 55753.53 ± 2127.46 |  | 56646.81 ± 3189.12 | 0.8623170 |
| *S100beta* | 20.65 ± 1.91 |  | 18.97 ± 3.88 | 0.5294030 |
| *Scn10a (Nav1.8)* | 12.57 ± 2.06 |  | 13.62 ± 1.29 | 0.5598157 |
| *Scn11a (Nav 1.9)* | 34.87 ± 3.54 |  | 29.51 ± 2.4 | 0.2432384 |
| *Scn8a* | 14.24 ± 1 |  | 15.14 ± 1.75 | 0.7733430 |
| *Scn9a (Nav1.7)* | 32.6 ± 2.26 |  | 30.32 ± 4.24 | 0.5532773 |
| *Semaphorin* | 169.38 ± 22.38 |  | 155.79 ± 18.92 | 0.6819627 |
| *Sox10* | 35.69 ± 5.57 |  | 35.45 ± 8.16 | 0.7420257 |
| *Subp* | 477.09 ± 61.38 |  | 477.09 ± 74.03 | 0.9358393 |
| *T-Bet* | 19.18 ± 4.44 |  | 12.05 ± 1.13 | 0.1335792 |
| *Taar1* | 9.46 ± 1.38 |  | 8.66 ± 1.17 | 0.6649720 |
| *Taar4* | 10.85 ± 0.95 |  | 13.91 ± 0.77 | 0.0525005 |
| *Tac1r* | 64.07 ± 11.13 |  | 55.15 ± 10.4 | 0.5822994 |
| *Tbp* | 396.15 ± 21.56 |  | 372.53 ± 14.24 | 0.4044394 |
| *Tff3* | 90668.06 ± 6915.55 |  | 80729.2 ± 5358.79 | 0.3036757 |
| *Tgfb* | 670.67 ± 122.54 |  | 473.04 ± 103.92 | 0.2459397 |
| *Tgr5* | 16.19 ± 0.89 |  | 17.05 ± 2.55 | 0.9100137 |
| *Tjp1* | 1815.19 ± 102.19 |  | 1804.26 ± 156.99 | 0.8945754 |
| *Tlr2* | 151.08 ± 7.92 |  | 150.74 ± 24.96 | 0.7910457 |
| *Tlr4* | 987.32 ± 94.32 |  | 1113.54 ± 57 | 0.2613525 |
| *Tlr5* | 96.64 ± 6.33 |  | 94.16 ± 13.73 | 0.7184652 |
| *Tnfa* | 63.55 ± 7.56 |  | 42.2 ± 5.19 | 0.0600386 |
| *Tollip* | 1252.05 ± 36.59 |  | 1261.81 ± 61.56 | 0.9384363 |
| *Trpa1* | 19.39 ± 2.92 |  | 21.51 ± 2.46 | 0.5526368 |
| *Trpc5* | 20.05 ± 3.06 |  | 17.91 ± 2.07 | 0.6506900 |
| *Trpm8* | 7.87 ± 0.52 |  | 7.64 ± 0.72 | 0.7387014 |
| *Trpv1* | 16.52 ± 2.59 |  | 17.93 ± 1.02 | 0.5167963 |
| *Trpv4* | 50.85 ± 4.6 |  | 50.76 ± 3.43 | 0.9734523 |
| *Vip* | 490.79 ± 48.17 |  | 461.78 ± 66.7 | 0.6575418 |
| *Vpac1* | 3451.97 ± 366.86 |  | 3137.09 ± 260.24 | 0.4980207 |
| *Vpac2* | 136.98 ± 27.96 |  | 126.69 ± 18.79 | 0.8630715 |
| Values are expressed as the mean ± SEM (n=5). Colonic gene expression was analysed using the NanoString platform. Data were analyzed by Wald test by NanoString nSolver 2.5. | | | | |
