# Supplementary figures and images for: Proteolytic bacteria expansion during colitis amplifies inflammation through cleavage of the external domain of PAR2
Source: Gut Microbes. 2024 Aug 22;16(1):2387857. doi: 10.1080/19490976.2024.2387857 (PMC11346554; doi:10.1080/19490976.2024.2387857)

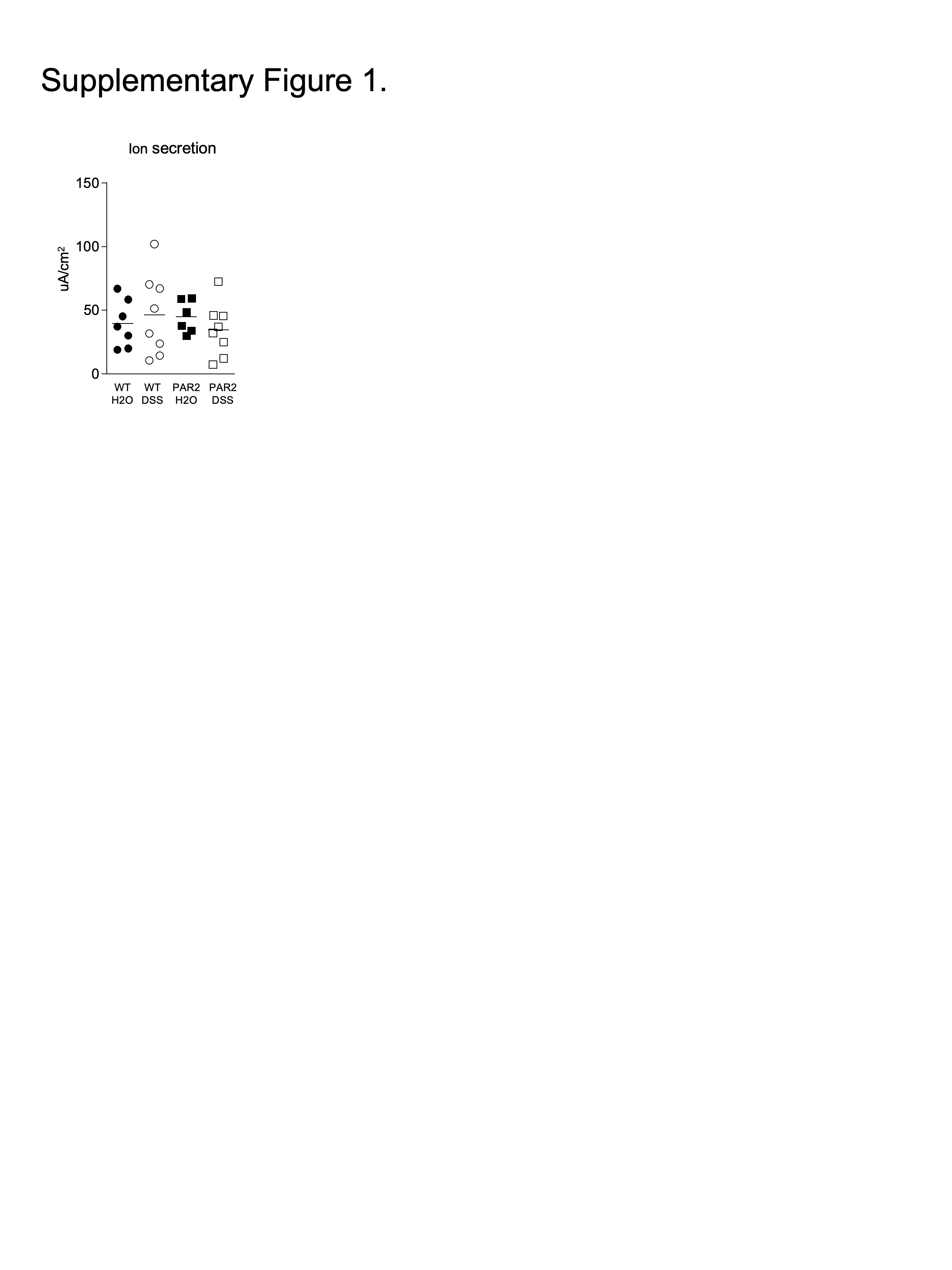

Supplement: Supplemental Material [file KGMI_A_2387857_SM7854.zip › Supplementary materials/Rondeau Figure S1.tiff]

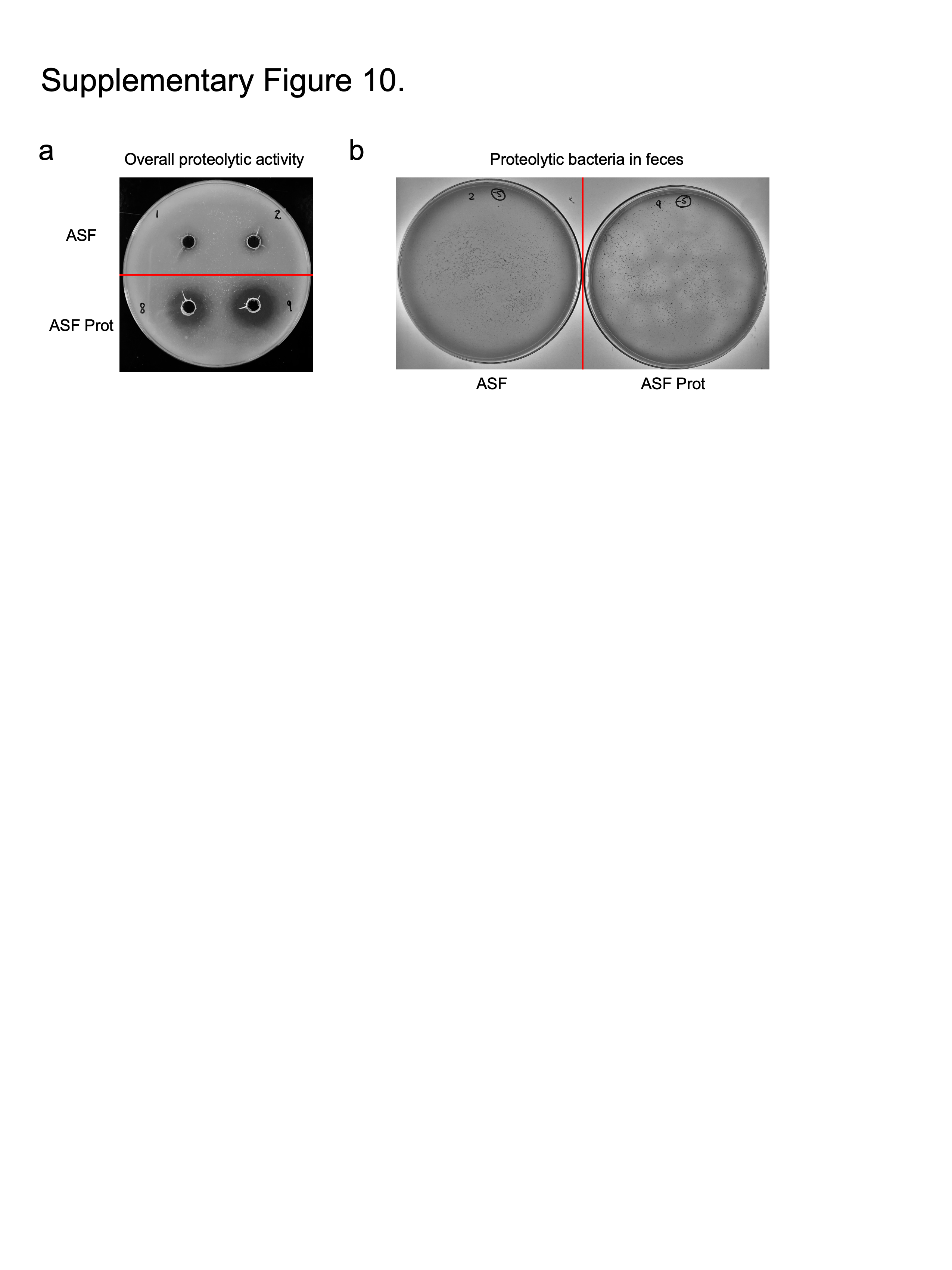

Supplement: Supplemental Material [file KGMI_A_2387857_SM7854.zip › Supplementary materials/Rondeau Figure S10.tiff]

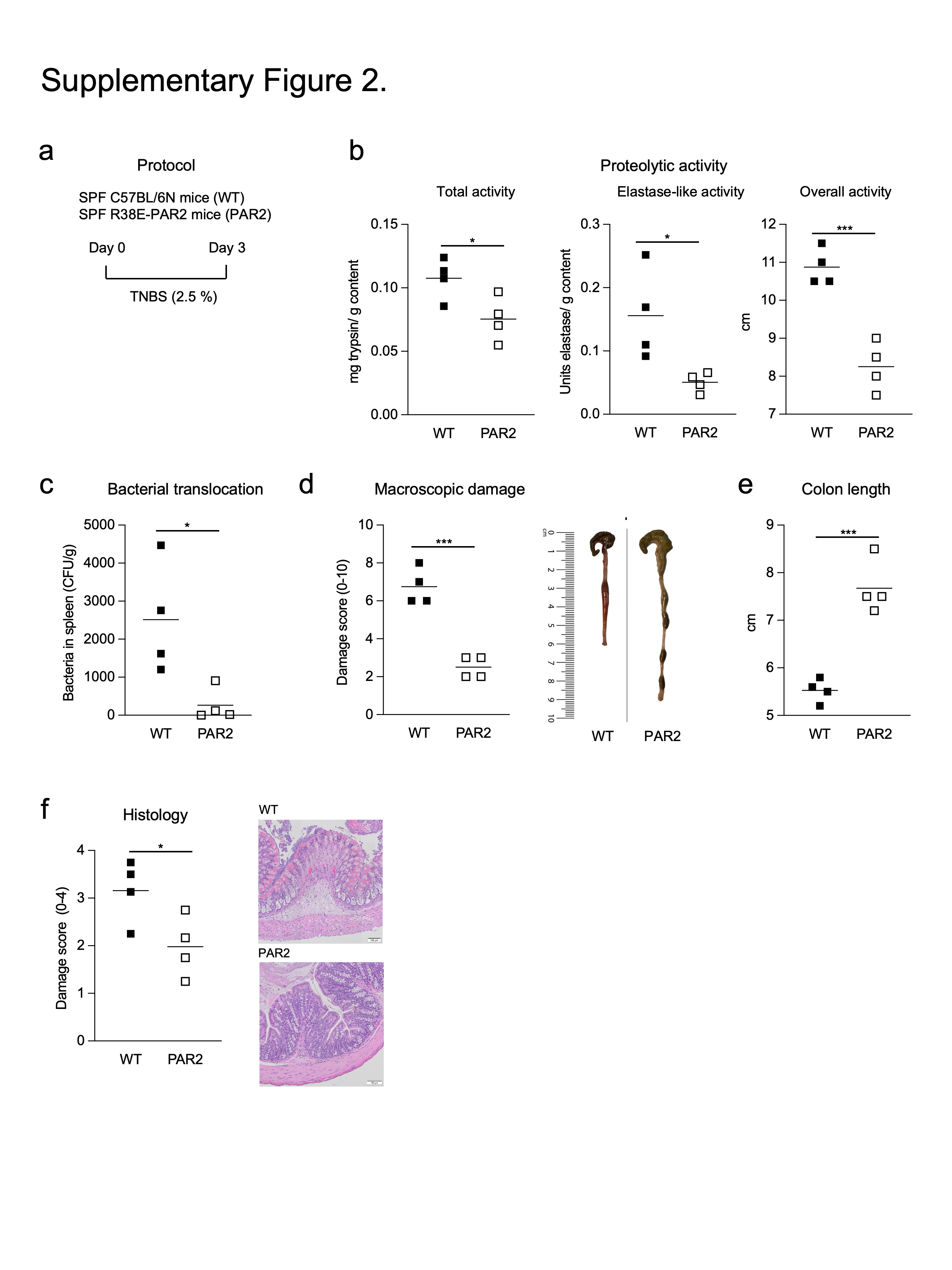

Supplement: Supplemental Material [file KGMI_A_2387857_SM7854.zip › Supplementary materials/Rondeau Figure S2.tiff]

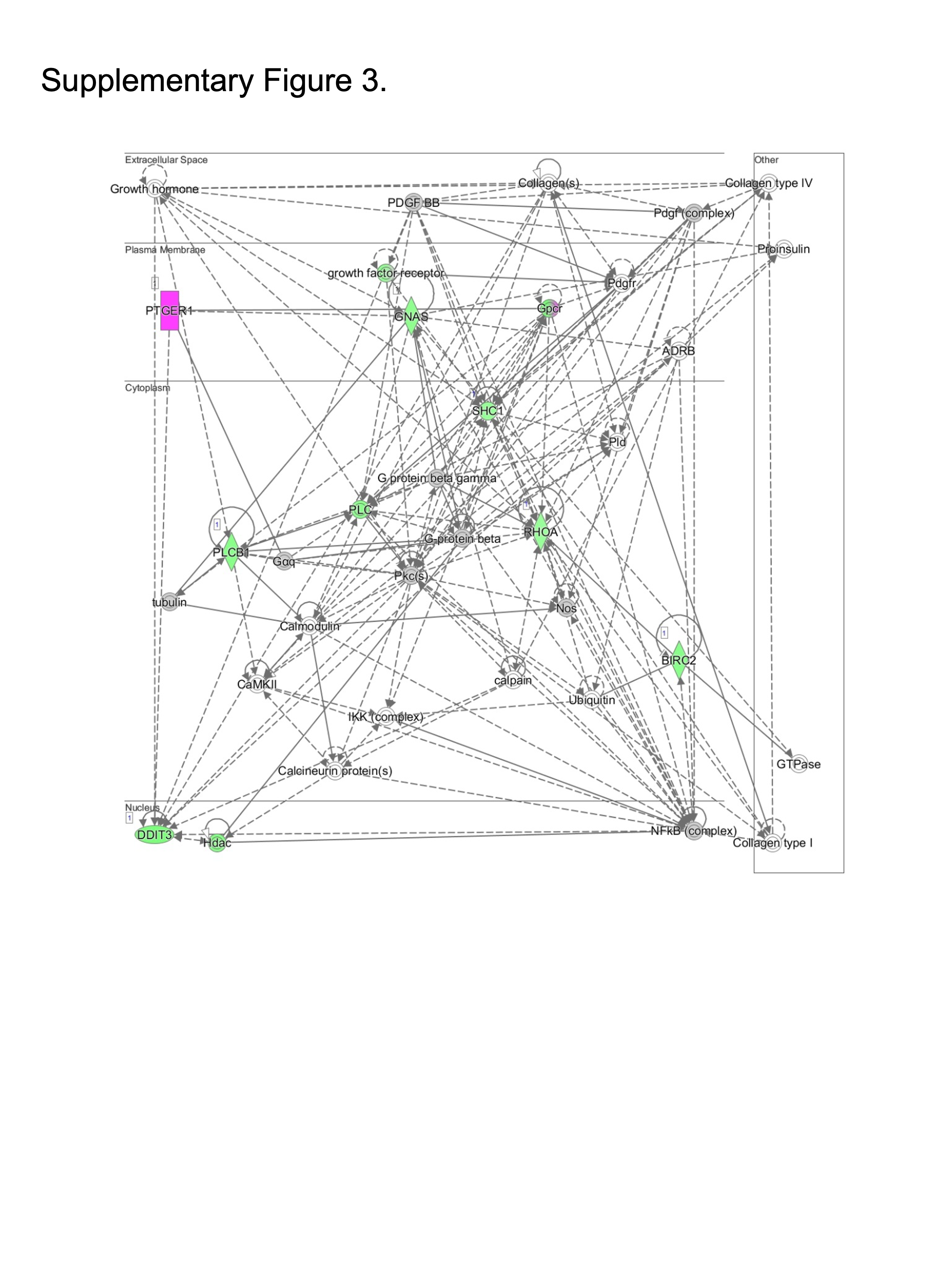

Supplement: Supplemental Material [file KGMI_A_2387857_SM7854.zip › Supplementary materials/Rondeau Figure S3.tiff]

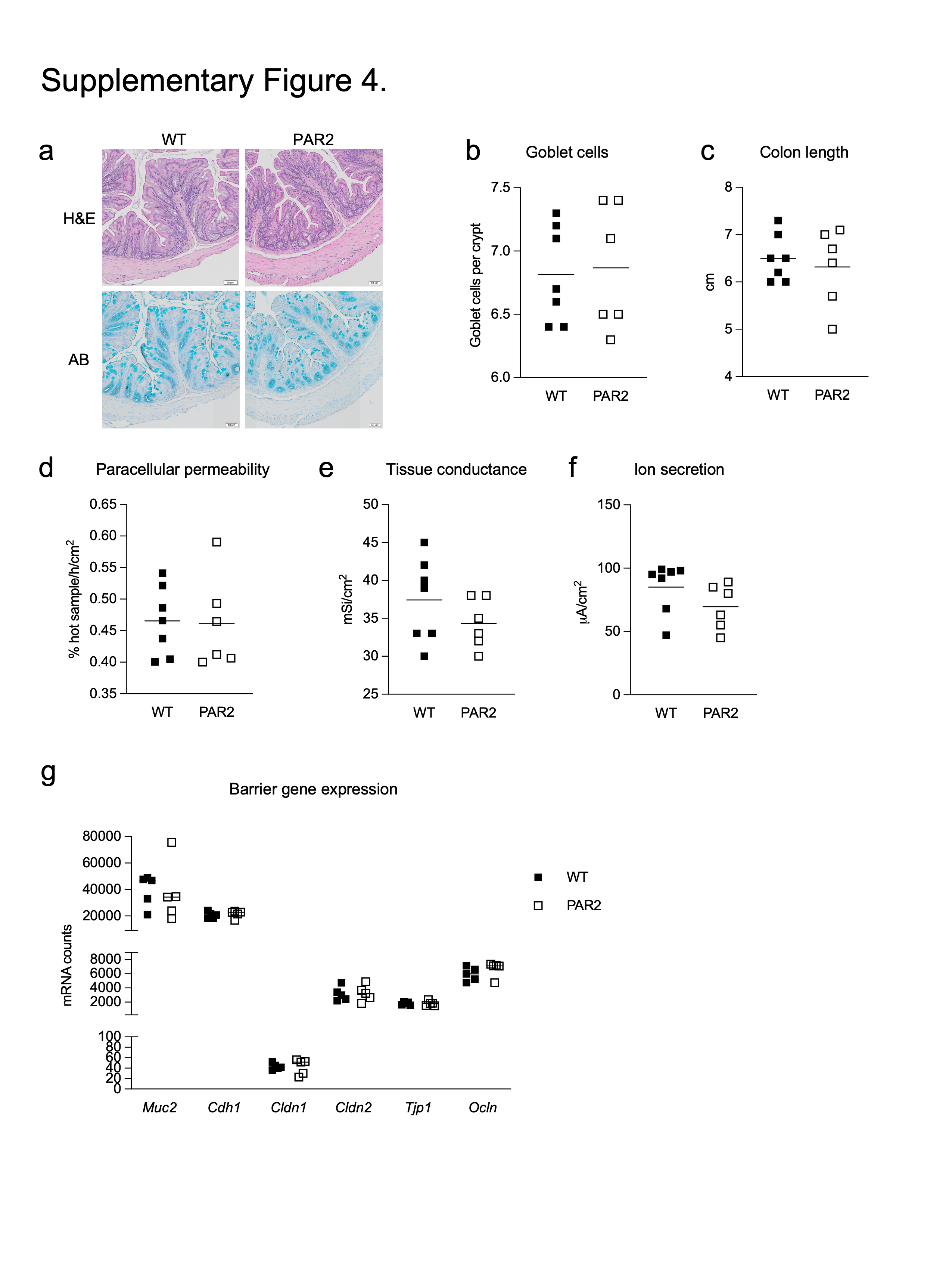

Supplement: Supplemental Material [file KGMI_A_2387857_SM7854.zip › Supplementary materials/Rondeau Figure S4.tiff]

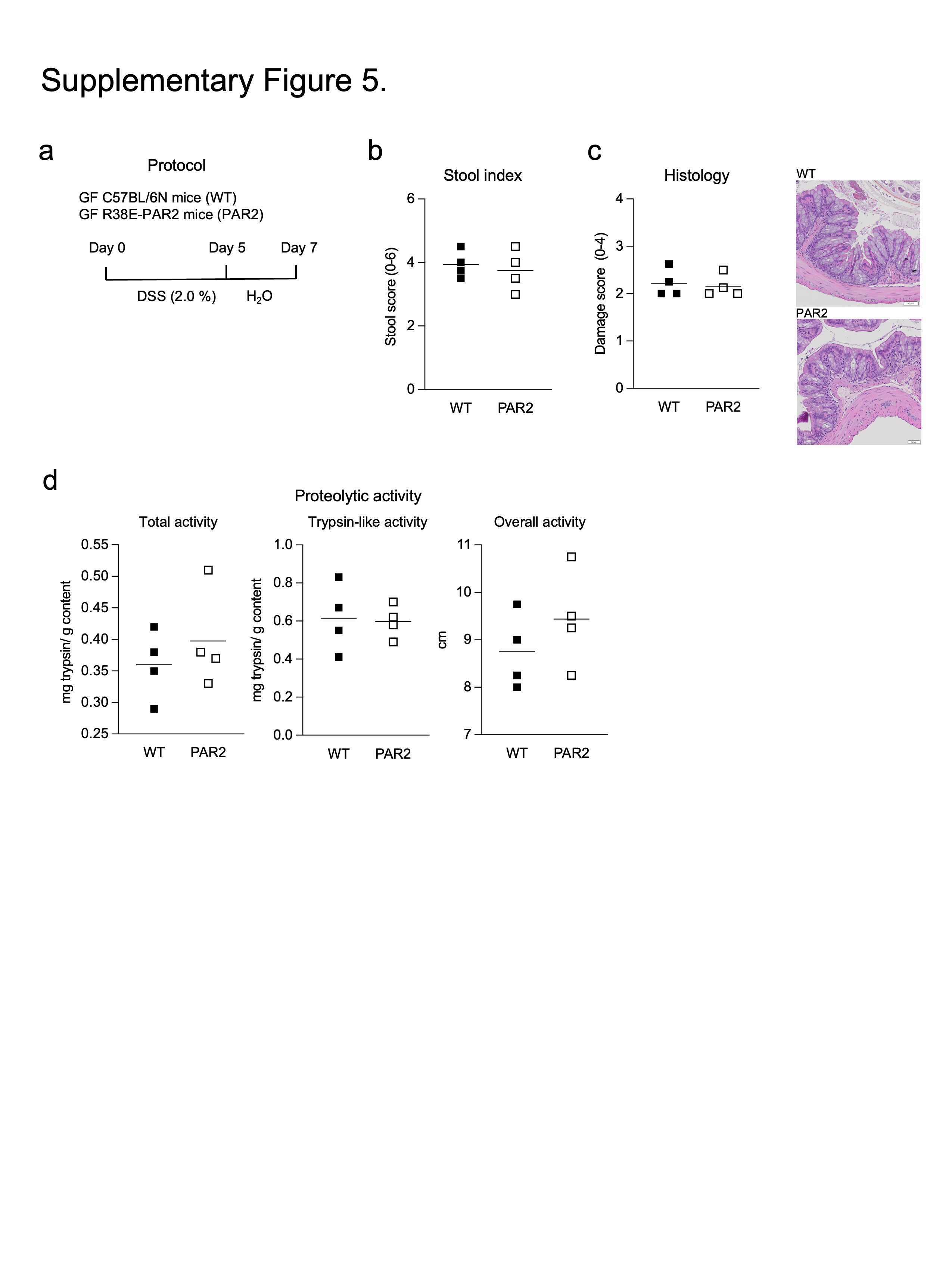

Supplement: Supplemental Material [file KGMI_A_2387857_SM7854.zip › Supplementary materials/Rondeau Figure S5.tiff]

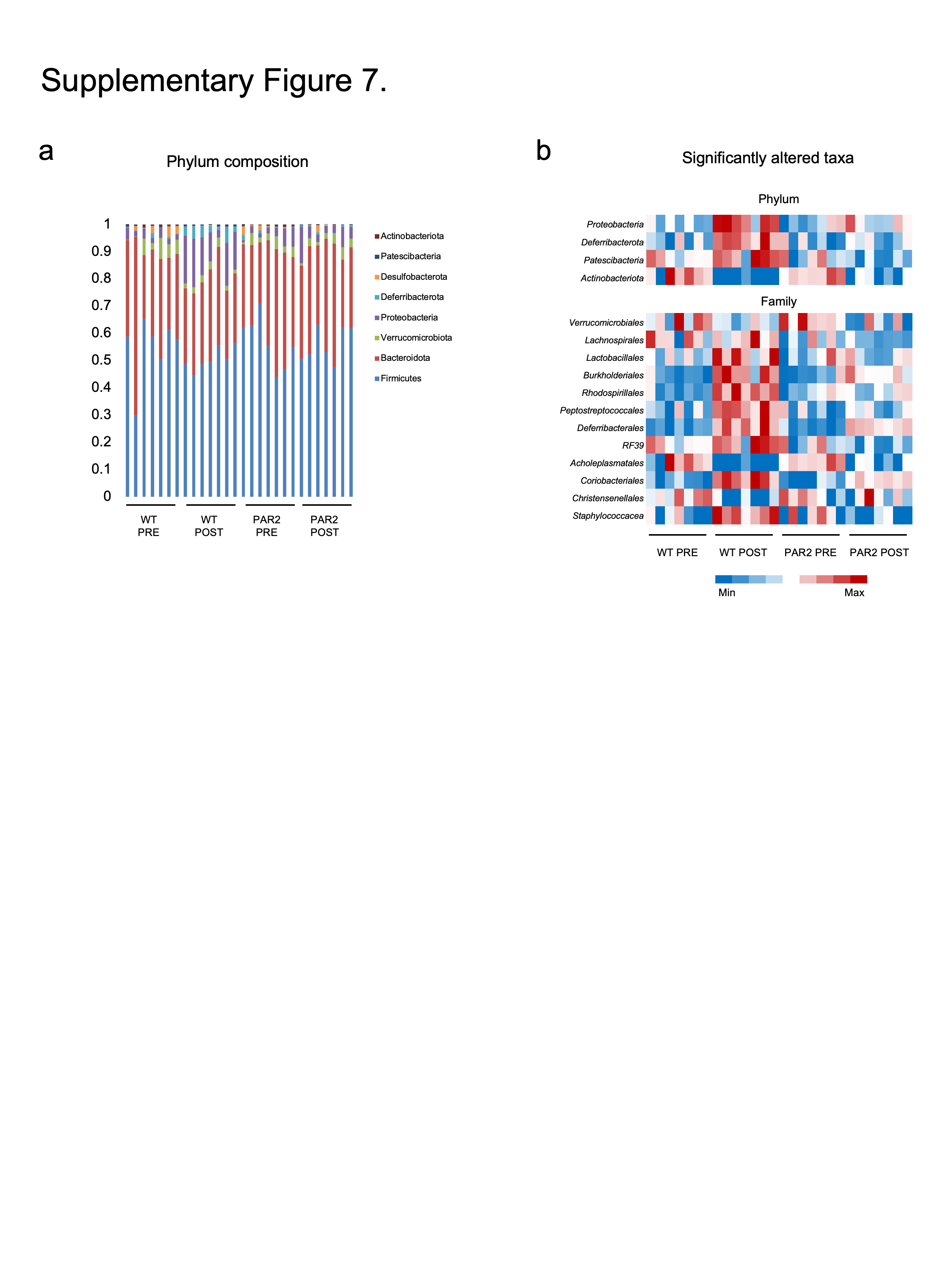

Supplement: Supplemental Material [file KGMI_A_2387857_SM7854.zip › Supplementary materials/Rondeau Figure S7.tiff]

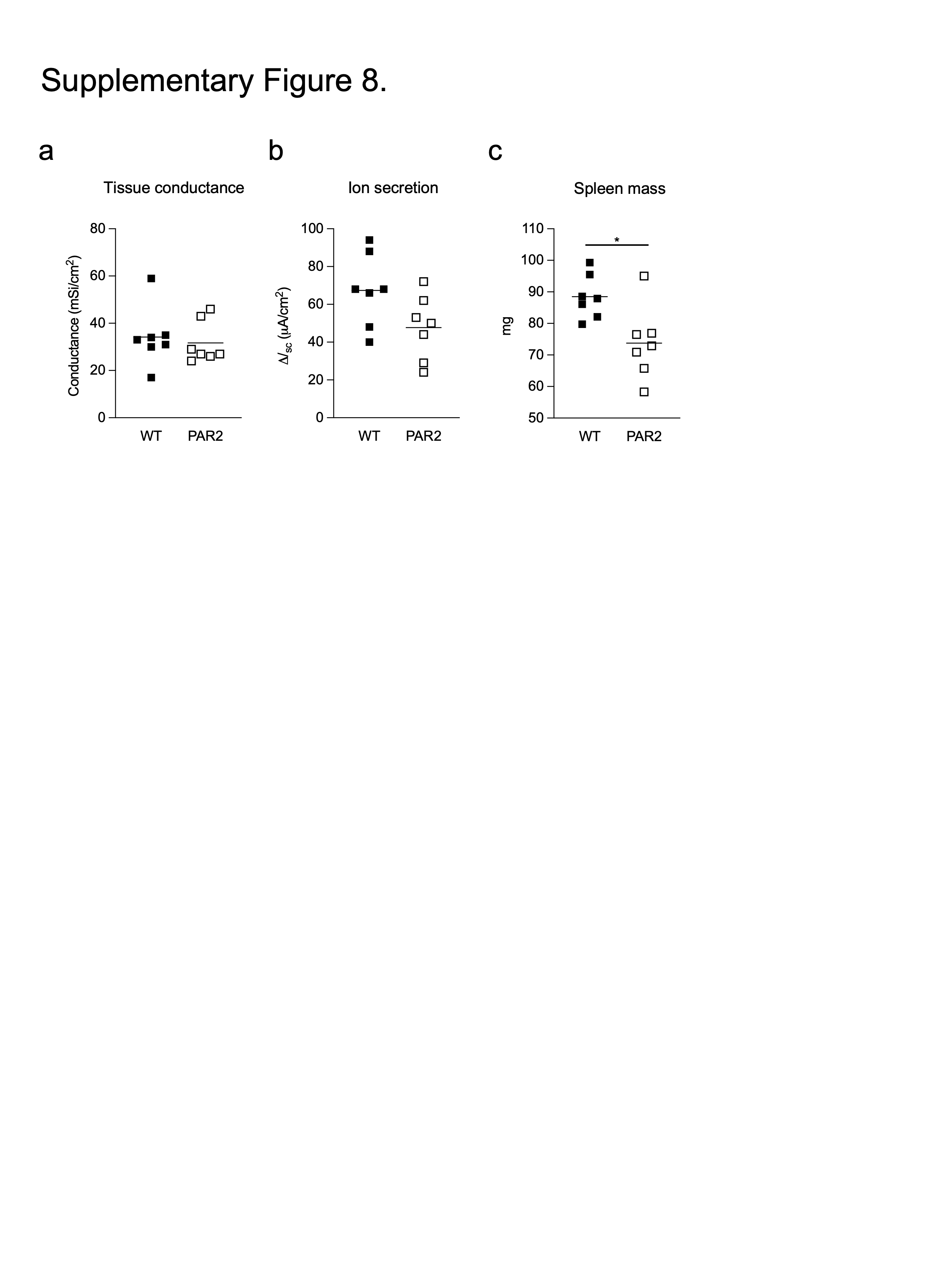

Supplement: Supplemental Material [file KGMI_A_2387857_SM7854.zip › Supplementary materials/Rondeau Figure S8.tiff]

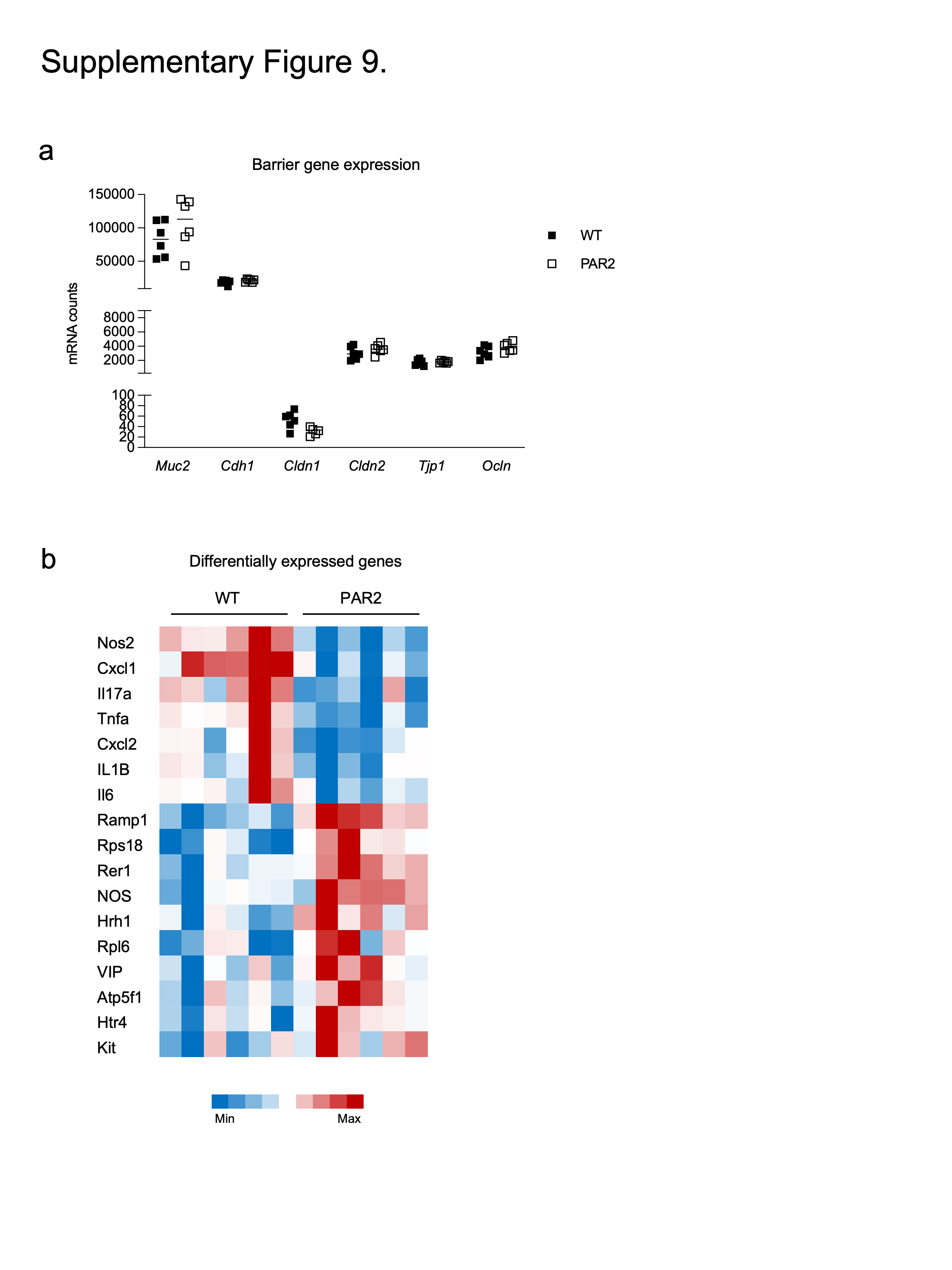

Supplement: Supplemental Material [file KGMI_A_2387857_SM7854.zip › Supplementary materials/Rondeau Figure S9.tiff]

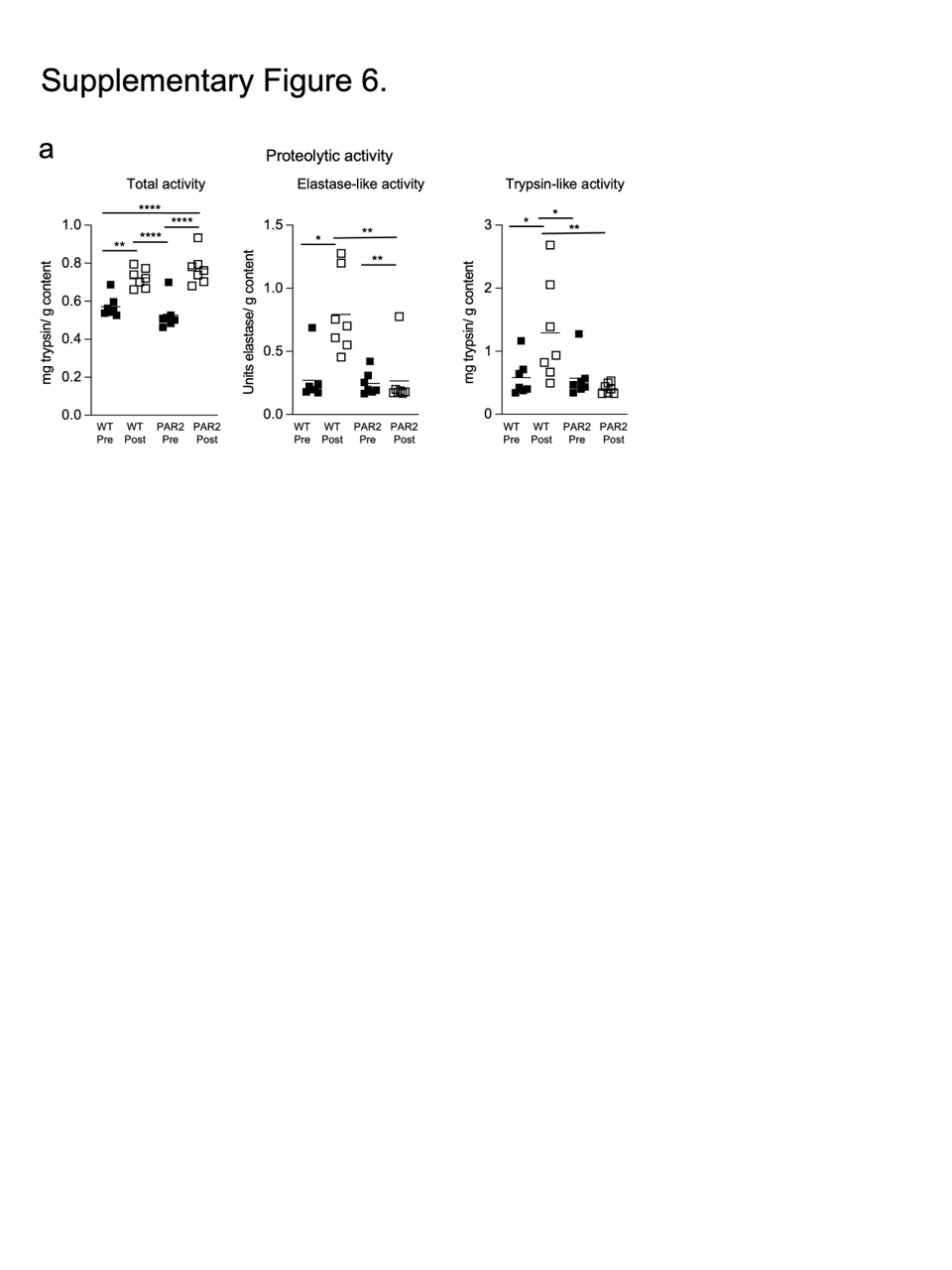

Supplement: Supplemental Material [file KGMI_A_2387857_SM7854.zip › Supplementary materials/Supplementary_Figure_6_2.tiff]
